# Comprehensive Analysis of Clinically Significant Hepatitis B Virus Mutations in Relation to Genotype, Subgenotype and Geographic Region

**DOI:** 10.3389/fmicb.2020.616023

**Published:** 2020-12-14

**Authors:** Natalia M. Araujo, Sheila A. Teles, Natália Spitz

**Affiliations:** ^1^Laboratory of Molecular Virology, Oswaldo Cruz Institute, FIOCRUZ, Rio de Janeiro, Brazil; ^2^Faculty of Nursing, Federal University of Goias, Goiânia, Brazil

**Keywords:** hepatitis B virus, genotypes, mutation, immune escape, antiviral resistance, hepatocellular carcinoma, HBV evolution, natural selection

## Abstract

Hepatitis B virus (HBV) is a highly variable DNA virus due to its unique life cycle, which involves an error-prone reverse transcriptase. The high substitution rate drives the evolution of HBV by generating genetic variants upon which selection operates. HBV mutants with clinical implications have been documented worldwide, indicating the potential for spreading and developing their own epidemiology. However, the prevalence of such mutants among the different HBV genotypes and subgenotypes has not been systematically analyzed. In the current study, we performed large-scale analysis of 6,479 full-length HBV genome sequences from genotypes A-H, with the aim of gaining comprehensive insights into the relationships of relevant mutations associated with immune escape, antiviral resistance and hepatocellular carcinoma (HCC) development with HBV (sub)genotypes and geographic regions. Immune escape mutations were detected in 10.7% of the sequences, the most common being I/T126S (1.8%), G145R (1.2%), M133T (1.2%), and Q129R (1.0%). HBV genotype B showed the highest rate of escape mutations (14.7%) while genotype H had no mutations (*P* < 0.001). HCC-associated mutations were detected in 33.7% of the sequences, with significantly higher frequency of C1653T, T1753V and A1762T/G1764A in genotype G than C (*P* < 0.001). The overall frequencies of lamivudine-, telbivudine-, adefovir-, and entecavir-resistant mutants were 7.3, 7.2, 0.5, and 0.2%, respectively, while only 0.05% showed reduced susceptibility to tenofovir. In particular, the highest frequency of lamivudine-resistant mutations was observed in genotype G and the lowest frequency in genotype E (32.5 and 0.3%; *P* < 0.001). The prevalence of HBV mutants was also biased by geographic location, with North America identified as one of the regions with the highest rates of immune escape, antiviral resistance, and HCC-associated mutants. The collective findings were discussed in light of natural selection and the known characteristics of HBV (sub)genotypes. Our data provide relevant information on the prevalence of clinically relevant HBV mutations, which may contribute to further improvement of diagnostic procedures, immunization programs, therapeutic protocols, and disease prognosis.

## Introduction

Hepatitis B is one of the most prevalent viral infections in humans and a major global public health problem. Hepatitis B virus (HBV) has infected one-third of the global population, with 257 million chronic infections worldwide and more than 880,000 deaths per year, mostly from cirrhosis and hepatocellular carcinoma (HCC) (WHO, 2020).

Hepatitis B virus is the prototype member of the family *Hepadnaviridae* containing a partially double-stranded relaxed circular DNA genome (∼3.2 kb) with a compact coding organization containing four partly or completely overlapping open reading frames (Seeger et al., 2013). Based on >7.5% genomic sequence divergence, HBV has been phylogenetically classified into nine genotypes (A–I) and one putative genotype (J). The significant diversity within specific HBV genotypes has led to further classification into numerous subgenotypes (Kramvis, 2014). HBV genotypes and subgenotypes have distinct geographic distributions. The unique replication cycle of HBV includes the activity of an error-prone reverse transcriptase (RT) that generates numerous viral variants (quasispecies). Constant HBV evolution leads to continued selection of mutations through pressure exerted by endogenous (host immune system) and exogenous (antiviral therapy and vaccination) factors, which fuels a molecular arms race between virus and host, resulting in emergence of mutations involved in several clinical effects (Araujo et al., 2011; Rajoriya et al., 2017; Revill et al., 2020).

The HBV surface antigen (HBsAg) contains a highly conserved antibody-neutralizing epitope cluster termed “a” determinant, which spans amino acids 124–147. Mutations occurring within or around this region lead to conformational changes, which can affect binding of neutralizing antibodies produced during natural infection or following active or passive immunization (Zuckerman and Zuckerman, 2003). These immune escape mutations account for several possible consequences, such as false-negative results by commercial HBsAg assays (occult hepatitis B) and evasion of anti-HBV immunoglobulin therapy and vaccine-induced immunity. G145R was the first escape mutation described and the most frequently detected HBV variant with proven vaccine escape properties in humans (Carman et al., 1990). Over the years, a number of other mutations associated with immune escape have been documented worldwide (Cooreman et al., 2001; Lazarevic et al., 2019).

Nucleos(t)ide analogs (NAs) are potent inhibitors of HBV RT activity. To date, six NAs, specifically, lamivudine (LAM), telbivudine (LdT), adefovir dipivoxil (ADV), entecavir (ETV), tenofovir disoproxil fumarate (TDF) and tenofovir alafenamide (TAF), have been approved for treatment of chronic HBV infections (Sarin et al., 2016; Easl, 2017; Terrault et al., 2018). However, drug-resistant mutations often arise during long-term use of therapies with a low barrier to resistance, such as LAM, leading to treatment failure and progression to liver disease (Zoulim, 2011). Additionally, owing to overlapping reading frames in the HBV genome, mutations in the polymerase gene resulting from antiviral selection pressure may affect neutralization epitopes within HBsAg (Pollicino et al., 2009).

The influence of HBV diversity on severity of liver disease has been investigated mainly for genotypes A-D (McMahon, 2009; Kao et al., 2010; Levrero and Zucman-Rossi, 2016). Genotype C is considered more oncogenic than genotypes A, B, and D (Chan et al., 2004; Wong et al., 2013). Likewise, mutations in the basal core promoter (BCP) resulting in decreased expression of HBeAg but enhanced viral replication and deletions in the Pre-S region are associated with increased risk of HCC (Liu et al., 2009).

The widespread distribution of HBV mutations with clinical implications poses a considerable challenge for design of diagnostic assays and current treatment strategies and is a potential threat to the long-term success of mass vaccination programs (Lazarevic, 2014; Yan et al., 2017). However, little is known on the frequencies of these mutations in different HBV genotypes. Knowledge of HBV subgenotypes is even more limited. Additionally, the unique distribution patterns of HBV (sub)genotypes in different geographic areas has hampered simultaneous comparisons. In the current study, we performed large-scale analysis of 6,479 full-length HBV genome sequences from genotypes A-H retrieved from public database with the aim of gaining comprehensive insights into the relationships of the most relevant escape-, resistance- and HCC-associated mutations with HBV (sub)genotypes and geographic regions. Knowledge of the prevalence of clinically relevant mutations among the different HBV genotypes and subgenotypes and their worldwide distribution should facilitate improvement of diagnostic procedures, immunization programs, therapeutic protocols, and disease prognosis.

## Materials and Methods

### HBV Genome Sequences

All complete genome sequences (*n* = 6,479) of HBV genotypes A to H from the Hepatitis B Virus Database (HBVdb) available at https://hbvdb.lyon.inserm.fr/HBVdb/HBVdbIndex (Hayer et al., 2013) were obtained. Precomputed datasets for each genotype containing sequences aligned in the Clustal W format were downloaded on the following dates: genotype A (*n* = 871), 18th September 2019; B (*n* = 1,755), 13th November 2019; C (*n* = 2,187), 14th January 2020; D (*n* = 1,059), 31st January 2020; E (*n* = 292), 17th February 2020; F (*n* = 249), G (*n* = 40), and H (*n* = 26), 18th February 2020. Information on the country of sequence origin was extracted from existing GenBank annotations. Sequences were further grouped into 21 geographic subregions according to the UN Statistics Division^1^.

### HBV Subgenotyping

Subgenotyping of all sequences was assessed via Maximum Likelihood (ML) phylogenetic analysis. ML phylogenetic tree was inferred with the IQ-TREE v.1.6.12 program (Nguyen et al., 2015) under the GTR + I + G nucleotide substitution model selected with ModelFinder (Kalyaanamoorthy et al., 2017). A heuristic tree search was performed with the aid of the nearest neighbor interchange (NNI) algorithm and the reliability of phylogeny estimated with the approximate likelihood-ratio test (Anisimova and Gascuel, 2006) based on a Shimodaira–Hasegawa-like procedure (SH-aLRT). We employed the newer proposals for subgenotype classification reviewed elsewhere (Kramvis, 2014). New classifications of subgenotypes proposed subsequently were not included in the analyses. GenBank accession numbers of the reference sequences are as follows: AB241115, AY233278 (subgenotype A1); AB116079, AY738141 (A2); AB194951, AB194952, AM180623, AY934764, FJ692609, FJ692613 (QS-A3); GQ331047, GQ331048 (A4); AB014366, D00329 (B1); AP011084, AY596111 (B2); AB219427, AP011085, AP011086, AP011091, AP011093, AP011094, EF473977, M54923 (QS-B3); AB073835, AB115551 (B4); AB287316, DQ463787 (B5); AB031265, AB112066 (C1); AB033553, AB368297, AF533983, HQ622095 (QS-C2); KU695745, KU695746, X75656, X75665 (C3); AB048704, AB048705, KF873543, KF873545 (C4); AB241109, AP011099, EU410081, KM999992 (C5); KM999993, AP011103, AP011102 (C6); EU670263 (C7); AP011104, AP011105, AP011106, AP011107 (C8); AP011108 (C9); AB540583 (C10); AB554019, AB554020 (C11); AB554018, AB554025 (C12); AB644280, AB644281 (C13); GQ377555, HM011493 (C14); AB644286 (C15); AB644287 (C16); FJ904424, X80926 (D1); AB109475, Z35716 (D2); AB493845, AB493846, AY233291, X65257 (D3); AB033559, AB048702 (D4); AB033558, DQ315779 (D5); AM494716, FJ904430 (D6); AB091256, HM363610, X75664 (E); AY090459, DQ823095, HM585194 (F1); AY090455, DQ899143, X69798 (F2); AB036905, AB036915, MH051986 (F3); AB166850, DQ823090, KJ843175 (F4); AB056513, AB064310, EF634480 (G); AB516395, AY090454, AY090457 (H); FJ023664, FJ023660 (I); AB486012 (J).

### Mutational Analysis

Sequence alignments were analyzed using MEGA version 10.1.8 (Kumar et al., 2018). An Excel database containing information on each nucleotide/amino acid in the respective positions of each sequence was constructed (Supplementary Table S1). A total of 21 clinically relevant positions according to previous reports were examined, including 10 amino acid positions within HBsAg (120, 126, 129, 130, 133, 141, 142, 143, 144, and 145) related to immune escape (Cooreman et al., 2001; Ma and Wang, 2012; Qin and Liao, 2018; Lazarevic et al., 2019), four nucleotide positions within the core promoter gene (1653 in enhancer II and 1753, 1762, and 1764 in BCP) and Pre-S deletions related to HCC development (Chou et al., 2008; Liu et al., 2009; Kao et al., 2010; Tseng et al., 2015), and seven amino acid positions within the RT domain of HBV polymerase (180, 181, 184, 202, 204, 236, and 250) related to antiviral resistance (Zoulim and Locarnini, 2009). According to the most recent clinical practice guidelines of the European Association for the Study of the Liver (Easl, 2017), the following amino acid substitution profiles for HBV-resistant mutants were used: rtM204V/I (LAM resistance), rtM204I or L180M + rtM204V (LdT resistance), rtA181T/V or rtN236T (ADV resistance), rtL180M + rtM204I/V ± rtT184S/G ± rtS202I/G ± rtM250V (ETV resistance), and rtA181T/V + rtN236T (TDF/TAF-reduced susceptibility).

### Statistical Analysis

Statistical analysis was performed using SPSS statistical software package version 23.0 (IBM SPSS Inc., Chicago, IL, United States). Frequencies were compared using the chi-squared test or Fisher’s exact test. *P*-values < 0.05 were considered statistically significant.

## Results

### HBV Subgenotypes and Geographic Distribution

**FIGURE 1:**
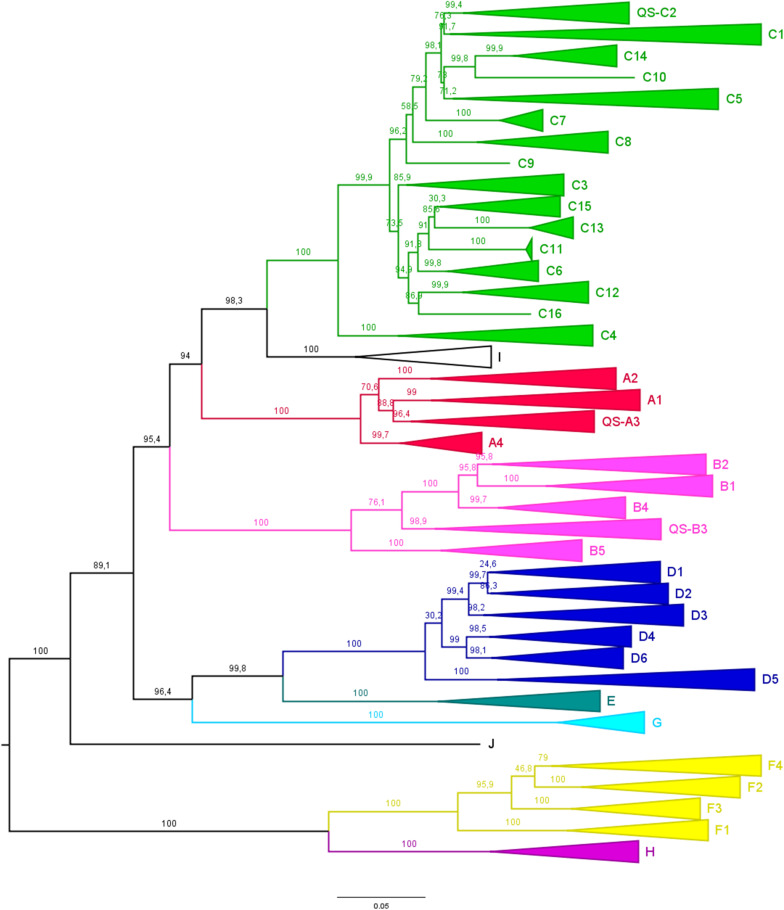
Phylogenetic analysis of HBV complete genome sequences representative of genotypes A–J. Maximum likelihood phylogenetic tree of 6,538 complete genome sequences (6,434 from HBVdb database plus 104 reference sequences). Sequences whose subgenotype could not be determined (*n* = 45) were excluded from the analysis. The branches are colored according to the genotypes. (Sub)genotypes are indicated next to their respective clusters. The numbers in branches indicate the statistical support (aLRT value).

**FIGURE 2:**
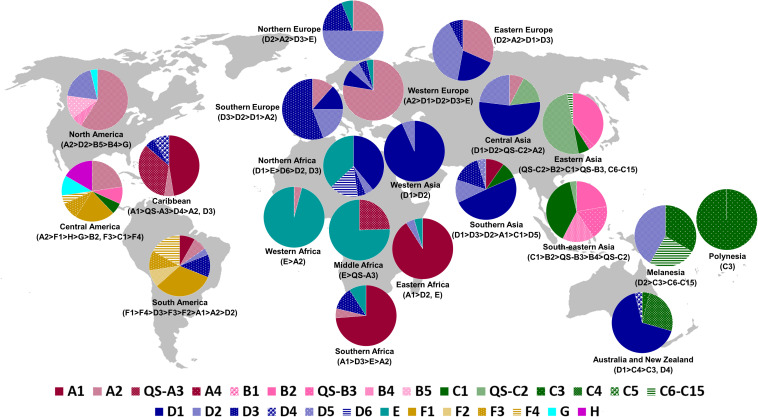
Geographic distribution of HBV genotypes and subgenotypes based on 6,137 complete genome sequences present in the HBVdb database. (Sub)genotypes with frequencies < 3% were not included in the figure. The map was reconstructed using Wikimedia Commons (https://commons.wikimedia.org/wiki/File:BlankMap-World-noborders.png), this figure is similar but not identical to the original image and is therefore for illustrative purposes only. QS, quasi-subgenotype.

For comprehensive evaluation, all HBV genome sequences from genotypes A, B, C, D, and F (*n* = 6,121) were classified into subgenotypes via phylogenetic analysis of each genotype dataset (Supplementary Figure S1). The phylogenetic tree of the complete data used in the study is shown in Figure 1. Subgenotyping results were further compared with information on subgenotypes of the sequences in GenBank, where available. Sequences with conflicting results (*n* = 29) or undetermined subgenotypes (*n* = 45) were not included in subsequent analyses. The following subgenotypes were identified for each genotype: A1 (*n* = 265, 30.4%), A2 (*n* = 553, 63.5%), QS-A3 (*n* = 48, 5.5%), A4 (*n* = 3, 0.3%), and not determined (*n* = 2, 0.2%) (genotype A, *n* = 871); B1 (*n* = 57, 3.2%), B2 (*n* = 1310, 74.6%), QS-B3 (*n* = 207, 11.8%), B4 (*n* = 128, 7.3%), B5 (*n* = 52, 3.0%), and not determined (*n* = 1, 0.1%) (genotype B, *n* = 1755); C1 (*n* = 451, 20.6%), QS-C2 (*n* = 1518, 69.4%), C3 (*n* = 28, 1.3%), C4 (*n* = 13, 0.6%), C5 (*n* = 19, 0.9%), C6–C15 (*n* = 103, 4.7%), not determined (*n* = 36, 1.6%) and conflicting results (*n* = 19, 0.9%) (genotype C, *n* = 2187); D1 (*n* = 516, 48.7%), D2 (*n* = 291, 27.5%), D3 (*n* = 189, 17.8%), D4 (*n* = 21, 2.0%), D5 (*n* = 21, 2.0%), D6 (*n* = 7, 0.7%), not determined (*n* = 6, 0.6%), and conflicting results (*n* = 8, 0.8%) (genotype D, *n* = 1059); F1 (*n* = 123, 49.4%), F2 (*n* = 29, 11.6%), F3 (*n* = 39, 15.7%), F4 (*n* = 56, 22.5%), and conflicting results (*n* = 2, 0.8%) (genotype F, *n* = 249). In addition, sequences with information on the country of origin available in GenBank were grouped into geographic subregions. For simplification purposes, sequences classified in subgenotypes C6–C15 were grouped as C6–C15. The global geographical distribution of HBV (sub)genotypes from 6,137 full-length genome sequences present in the HBVdb database is shown in Figure 2. The sequence list and corresponding background information is available as Supplementary Table S1.

### Immune escape Mutations

Ten positions within HBsAg (P120, I/T126, Q129, G130, M133, K141, P142, S/T143, D144, and G145) incorporating 25 immune escape mutations were analyzed. The amino acid frequencies at each position in different HBV genotypes are shown in Table 1. Overall, immune escape mutations were detected in 691 of 6,479 sequences (10.7%). HBV genotype B showed the highest frequency of mutation (14.7%), followed by genotypes C (11.2%), G (10%), D (9.3%), A (7.5%), E (5.1%), and F (2.0%) (*P* < 0.001). No escape mutations were detected in genotype H sequences. The most common substitutions were I/T126S (1.8%), G145R (1.2%), M133T (1.2%), Q129R (1.0%), I/T126A (0.8%), and P120T (0.8%). These major mutations were more frequent in genotypes A (P120T, 3.2%), B (I/T126A, 2.8%; Q129R, 2.2%), C (I/T126S, 3.9%; M133T, 1.9%), and G (G145R, 5.0%). Notably, G145R was only detected in genotypes G (5.0%), D (2.5%), C (2.1%), and B (0.2%) (*P* < 0.001) (Table 1).

**TABLE 1:** Clinically relevant HBV mutations by genotype.

	Genotypes, n (%)										
Sites	Total (*n* = 6479)	A (*n* = 871)	B (*n* = 1755)	C (*n* = 2187)	D (*n* = 1059)	E (*n* = 292)	F (*n* = 249)	G (*n* = 40)	H (*n* = 26)	Clinical issue	*P*
P120										Immune escape	
L	2 (0.0)	0	0	0	1 (0.1)	1 (0.3)	0	0	0		0.096
Q	3 (0.0)	0	1 (0.1)	0	1 (0.1)	0	1 (0.4)	0	0		0.256
S	43 (0.7)	1 (0.1)	25 (1.4)	2 (0.1)	14 (1.3)	1 (0.3)	0	0	0		<0.001
T	50 (0.8)	28 (3.2)	5 (0.3)	8 (0.4)	9 (0.8)	0	0	0	0		<0.001
Total	98 (1.5)	29 (3.3)	31 (1.8)	10 (0.5)	25 (2.8)	2 (0.7)	1 (0.4)	0	0		<0.000
I/T126										Immune escape	
A	53 (0.8)	1 (0.1)	50 (2.8)	0	0	0	2 (0.8)	0	0		<0.001
N	24 (0.4)	0	1 (0.1)	21 (1.0)	2 (0.2)	0	0	0	0		<0.001
S	116 (1.8)	14 (1.6)	14 (0.8)	86 (3.9)	2 (0.2)	0	0	0	0		<0.001
Total	193 (3.0)	15 (1.7)	65 (3.7)	107 (4.9)	4 (0.4)	0	2 (0.8)	0	0		<0.001
Q129										Immune escape	
H	46 (0.7)	0	39 (2.2)	2 (0.1)	4 (0.4)	1 (0.3)	0	0	0		<0.001
N	4 (0.1)	0	0	4 (0.2)	0	0	0	0	0		0.346
R	63 (1.0)	4 (0.5)	39 (2.2)	6 (0.3)	14 (1.3)	0	0	0	0		<0.001
Total	113 (1.7)	4 (0.5)	78 (4.4)	12 (0.5)	18 (1.7)	1 (0.3)	0	0	0		<0.001
G130										Immune escape	
N	36 (0.6)	4 (0.5)	4 (0.2)	25 (1.1)	3 (0.3)	0	0	0	0		0.003
R	11 (0.2)	0	4 (0.2)	5 (0.2)	0	1 (0.3)	1 (0.4)	0	0		0.600
Total	47 (0.7)	4 (0.5)	8 (0.5)	30 (1.4)	3 (0.3)	1 (0.3)	1 (0.4)	0	0		0.006
M133										Immune escape	
I	16 (0.2)	3 (0.3)	4 (0.2)	3 (0.1)	3 (0.3)	3 (1.0)	0	0	0		0.219
L	47 (0.7)	0	43 (2.5)	2 (0.1)	1 (0.1)	1 (0.3)	0	0	0		<0.001
T	79 (1.2)	8 (0.9)	26 (1.5)	42 (1.9)	1 (0.1)	2 (0.7)	0	0	0		<0.001
Total	142 (2.2)	11 (1.3)	73 (4.2)	47 (2.1)	5 (0.5)	6 (2.1)	0	0	0		<0.001
K141										Immune escape	
E	3 (0.0)	1 (0.1)	1 (0.1)	1 (0.0)	0	0	0	0	0		0.975
I	2 (0.0)	0	0	2 (0.1)	0	0	0	0	0		0.788
Total	5 (0.1)	1 (0.1)	1 (0.1)	3 (0.1)	0	0	0	0	0		0.922
P142										Immune escape	
L	4 (0.1)	0	0	1 (0.0)	2 (0.2)	0	0	1 (2.5)	0		<0.001
S	2 (0.0)	1 (0.1)	1 (0.1)	0	0	0	0	0	0		0.828
Total	6 (0.1)	1 (0.1)	1 (0.1)	1 (0.0)	2 (0.2)	0	0	1 (2.5)	0		<0.001
S/T143L	22 (0.3)	0	0	1 (0.0)	20 (1.9)	1 (0.3)	0	0	0	Immune escape	<0.001
D144										Immune escape	
A	4 (0.1)	2 (0.2)	0	1 (0.0)	0	1 (0.3)	0	0	0		0.204
E	13 (0.2)	2 (0.2)	1 (0.1)	2 (0.1)	4 (0.4)	2 (0.7)	1 (0.4)	1 (2.5)	0		0.007
G	3 (0.0)	1 (0.1)	0	1 (0.0)	1 (0.1)	0	0	0	0		0.927
Total	20 (0.3)	5 (0.6)	1 (0.1)	4 (0.2)	5 (0.5)	3 (1.0)	1 (0.4)	1 (2.5)	0		0.008
G145										Immune escape	
A	34 (0.5)	1 (0.1)	4 (0.2)	26 (1.2)	2 (0.2)	1 (0.3)	0	0	0		<0.001
R	78 (1.2)	0	4 (0.2)	46 (2.1)	26 (2.5)	0	0	2 (5.0)	0		<0.001
Total	112 (1.7)	1 (0.1)	8 (0.5)	72 (3.3)	28 (2.6)	1 (0.3)	0	2 (5.0)	0		<0.001
Escape	691 (10.7)	65 (7.5)	258 (14.7)	245 (11.2)	99 (9.3)	15 (5.1)	5 (2.0)	4 (10)	0	Immune escape	<0.001
mutants^*a*^											
C1653T	474 (7.3)	28 (3.2)	28 (1.6)	262 (12)	93 (8.8)	16 (5.5)	7 (2.8)	38 (95)	2 (7.7)	HCC	<0.001
T1753V	820 (12.7)	60 (6.9)	78 (4.4)	395 (18.1)	176 (16.6)	40 (13.7)	32 (12.9)	38 (95)	1 (3.8)	HCC	<0.001
A1762T/G1764A	1871 (28.9)	234 (26.9)	272 (15.5)	1009 (46.1)	228 (21.5)	32 (11)	56 (22.5)	39 (97.5)	1 (3.8)	HCC	<0.001
Pre-S deletions	98 (1.5)	10 (1.1)	10 (0.6)	73 (3.3)	0	2 (0.7)	3 (1.2)	0	0	HCC	<0.001
HCC-	2183 (33.7)	260 (29.9)	318 (18.1)	1088 (49.7)	327 (30.9)	71 (24.3)	76 (30.5)	39 (97.5)	4 (15.4)	HCC	<0.001
associated											
mutants^*b*^											
rtL180M	309 (4.8)	121 (13.9)	24 (1.4)	90 (4.1)	60 (5.7)	1 (0.3)	8 (3.2)	3 (7.5)	2 (7.7)	Antiviral resistance	<0.001
rtA181										Antiviral resistance	
T	3 (0.0)	0	0	3 (0.1)	0	0	0	0	0		0.553
V	18 (0.3)	0	2 (0.1)	15 (0.7)	1 (0.1)	0	0	0	0		0.005
Total	21 (0.3)	0	2 (0.1)	18 (0.8)	1 (0.1)	0	0	0	0		0.001
rtT184										Antiviral resistance	
G	0	0	0	0	0	0	0	0	0		–
S	2 (0.0)	0	0	1 (0.0)	1 (0.1)	0	0	0	0		0.924
Total	2 (0.0)	0	0	1 (0.0)	1 (0.1)	0	0	0	0		0.924
rtS202										Antiviral resistance	
G	11 (0.2)	0	3 (0.2)	6 (0.3)	1 (0.1)	0	0	0	1 (3.8)		0.001
I	1 (0.0)	0	0	0	1 (0.1)	0	0	0	0		0.645
Total	12 (0.2)	0	3 (0.2)	6 (0.3)	2 (0.2)	0	0	0	1 (3.8)		0.002
rtM204										Antiviral resistance	
I	237 (3.7)	9 (1.0)	41 (2.3)	125 (5.7)	51 (4.8)	0	1 (0.4)	10 (25)	0		<0.001
V	236 (3.6)	114 (13.1)	20 (1.1)	62 (2.8)	27 (2.5)	1 (0.3)	7 (2.8)	3 (7.5)	2 (7.7)		<0.001
Total	472 (7.3)	123 (14.1)	61 (3.5)	187 (8.6)	78 (7.4)	1 (0.3)	8 (3.2)	13 (32.5)	2 (7.7)		<0.001
rtN236T	17 (0.3)	1 (0.1)	12 (0.7)	4 (0.2)	0	0	0	0	0	Antiviral resistance	0.014
rtM250V	3 (0.0)	0	0	3 (0.1)	0	0	0	0	0	Antiviral resistance	0.053
LAM-resistant mutants^*c*^	473 (7.3)	123 (14.1)	61 (3.5)	187 (8.6)	78 (7.4)	1 (0.3)	8 (3.2)	13 (32.5)	2 (7.7)	Antiviral resistance	<0.001
LdT-resistant mutants^*d*^	466 (7.2)	120 (13.8)	61 (3.5)	183 (8.4)	78 (7.4)	1 (0.3)	8 (3.2)	13 (32.5)	2 (7.7)	Antiviral resistance	<0.001
ADV-resistant mutants^*e*^	35 (0.5)	1 (0.1)	13 (0.7)	20 (0.9)	1(0.1)	0	0	0	0	Antiviral resistance	0.016
ETV-resistant mutants^*f*^	11 (0.2)	0	0	8 (0.4)	2 (0.2)	0	0	0	1 (3.8)	Antiviral resistance	<0.001
TDF/TAF-reduced susceptibility mutants^*g*^	3 (0.0)	0	1 (0.1)	2 (0.1)	0	0	0	0		Antiviral resistance	0.949

*The P-value was listed as “–” when the specific mutation was found in no genotype (statistical analysis not conducted). LAM, lamivudine; LdT, telbivudine; ADV, adefovir; ETV, entecavir; TDF, tenofovir disoproxil fumarate; TAF, tenofovir alafenamide; rt, reverse transcriptase. ^a^Sequences with at least one immune escape mutation. ^b^Sequences with at least one HCC-associated mutation. ^c^Amino acid substitution profile for LAM resistance: rtM204V/I. ^d^Amino acid substitution profile for LdT resistance: rtM204I or L180M + M204V. ^e^Amino acid substitution profile for ADV resistance: rtA181T/V or rtN236T. ^f^Amino acid substitution profile for ETV resistance: rtL180M + rtM204I/V ± rtT184S/G ± rtS202I/G ± rtM250V. ^g^Amino acid substitution profile for TDF/TAF-reduced susceptibility: rtA181T/V + rtN236T.*

The HBV subgenotypes A2, B1, C6–C15, D4 and F2 displayed the highest frequencies of immune escape mutants within their respective genotypes (A2: 9.6%; B1: 15.8%; C6-C15: 36.9%; D4: 19%; F2: 6.9%), with significant differences within genotypes A and C (*P* = 0.018 and *P* < 0.001, respectively). On the other hand, subgenotypes A4, C4, C5, and D6 had no mutations in the listed HBsAg positions. In particular, significant differences in the frequency of G145R were observed within genotypes C and D, with the highest rates recorded for subgenotypes C6–C15 and D2 (34 and 8.2%, *P* < 0.001 for both; Tables 2–6).

**TABLE 2:** Clinically relevant mutations in HBV/A subgenotypes.

	Subgenotypes, n (%)					
Sites	A1 (*n* = 265)	A2 (*n* = 553)	QS-A3 (*n* = 48)	A4 (*n* = 3)	Clinical issue	*P*
P120					Immune escape	
L	0	0	0	0		–
Q	0	0	0	0		–
S	0	1 (0.2)	0	0		0.903
T	2 (0.8)	26 (4.7)	0	0		0.013
Total	2 (0.8)	27 (4.9)	0	0		0.010
I/T126					Immune escape	
A	1 (0.4)	0	0	0		0.516
N	0	0	0	0		–
S	1 (0.4)	13 (2.4)	0	0		0.152
Total	2 (0.8)	13 (2.4)	0	0		0.303
Q129					Immune escape	
H	0	0	0	0		–
N	0	0	0	0		–
R	2 (0.8)	2 (0.4)	0	0		0.836
Total	2 (0.8)	2 (0.4)	0	0		0.836
G130					Immune escape	
N	3 (1.1)	1 (0.2)	0	0		0.285
R	0	0	0	0		–
Total	3 (1.1)	1 (0.2)	0	0		0.285
M133					Immune escape	
I	2 (0.8)	0	1 (2.1)	0		0.059
L	0	0	0	0		–
T	1 (0.4)	7 (1.3)	0	0		0.561
Total	3 (1.1)	7 (1.3)	1 (2.1)	0		0.954
K141					Immune escape	
E	0	1 (0.2)	0	0		0.903
I	0	0	0	0		–
Total	0	1 (0.2)	0	0		0.903
P142					Immune escape	
L	0	0	0	0		–
S	0	1 (0.2)	0	0		0.903
Total	0	1 (0.2)	0	0		0.903
S/T143L	0	0	0	0	Immune escape	–
D144					Immune escape	
A	1 (0.4)	1 (0.2)	0	0		0.935
E	0	2 (0.4)	0	0		0.766
G	1 (0.4)	0	0	0		0.516
Total	2 (0.8)	3 (0.5)	0	0		0.926
G145					Immune escape	
A	0	1 (0.2)	0	0		0.903
R	0	0	0	0		–
Total	0	1 (0.2)	0	0		0.903
Escape mutants^*a*^	11 (4.2)	53 (9.6)	1 (2.1)	0	Immune escape	0.018
C1653T	14 (5.3)	8 (1.4)	6 (12.5)	0	HCC	<0.001
T1753V	24 (9.1)	28 (5.1)	7 (14.6)	1 (33.3)	HCC	<0.006
A1762T/G1764A	70 (26.4)	148 (26.8)	15 (31.3)	1 (33.3)	HCC	0.905
Pre-S deletions	8 (3.0)	2 (0.4)	0	0	HCC	0.008
HCC-associated mutants^*b*^	85 (32.1)	157 (28.4)	17 (35.4)	1 (33.3)	HCC	0.591
rtL180M	2 (0.8)	119 (21.5)	0	0	Antiviral resistance	<0.001
rtA181					Antiviral resistance	
T	0	0	0	0		–
V	0	0	0	0		–
rtT184					Antiviral resistance	
G	0	0	0	0		–
S	0	0	0	0		–
rtS202					Antiviral resistance	
G	0	0	0	0		–
I	0	0	0	0		–
rtM204					Antiviral resistance	
I	0	9 (1.6)	0	0		0.158
V	4 (1.5)	110 (19.9)	0	0		<0.001
Total	4 (1.5)	119 (21.5)	0	0		<0.001
rtN236T	0	1 (0.2)	0	0	Antiviral resistance	0.903
rtM250V	0	0	0	0	Antiviral resistance	–
LAM-resistant mutants^*c*^	4 (1.5)	119 (21.5)	0	0	Antiviral resistance	<0.001
LdT-resistant mutants^*d*^	2 (0.8)	118 (21.3)	0	0	Antiviral resistance	<0.001
ADV-resistant mutants^*e*^	0	1 (0.2)	0	0	Antiviral resistance	0.903
ETV-resistant mutants^*f*^	0	0	0	0	Antiviral resistance	–
TDF/TAF-reduced susceptibility mutants^*g*^	0	0	0	0	Antiviral resistance	–

*The P-value was listed as “–” when the specific mutation was found in no genotype (statistical analysis not conducted). LAM, lamivudine; LdT, telbivudine; ADV, adefovir; ETV, entecavir; TDF, tenofovir disoproxil fumarate; TAF, tenofovir alafenamide; QS, quasi-subgenotype. ^a^Sequences with at least one immune escape mutation. ^b^Sequences with at least one HCC-associated mutation. ^c^Amino acid substitution profile for LAM resistance: rtM204V/I. ^d^Amino acid substitution profile for LdT resistance: rtM204I or L180M + M204V. ^e^Amino acid substitution profile for ADV resistance: rtA181T/V or rtN236T. ^f^Amino acid substitution profile for ETV resistance: rtL180M + rtM204I/V ± rtT184S/G ± rtS202I/G ± rtM250V. ^g^Amino acid substitution profile for TDF/TAF-reduced susceptibility: rtA181T/V + rtN236T.*

**TABLE 3:** Clinically relevant mutations in HBV/B subgenotypes.

	Subgenotypes, n (%)						
Sites	B1 (*n* = 57)	B2 (*n* = 1310)	QS-B3 (*n* = 207)	B4 (*n* = 128)	B5 (*n* = 52)	Clinical issue	*P*
P120						Immune escape	
L	0	0	0	0	0		−
Q	0	1 (0.1)	0	0	0		0.987
S	1 (1.8)	20 (1.5)	2 (1.0)	2 (1.6)	0		0.875
T	0	4 (0.3)	0	1 (0.8)	0		0.730
Total	1 (1.8)	25 (1.9)	2 (1.0)	3 (2.3)	0		0.718
I/T126						Immune escape	
A	4 (7.0)	46 (3.5)	0	0	0		0.002
N	0	0	0	1 (0.8)	0		0.013
S	1 (1.8)	12 (0.9)	0	1 (0.8)	0		0.562
Total	5 (8.8)	58 (4.4)	0	2 (1.6)	0		0.01
Q129						Immune escape	
H	0	35 (2.7)	4 (1.9)	0	0		0.154
N	0	0	0	0	0		–
R	1 (1.8)	35 (2.7)	0	3 (2.3)	0		0.127
Total	1 (1.8)	35 (2.7)	4 (1.9)	3 (2.3)	0		0.036
G130						Immune escape	
N	1 (1.8)	0	0	2 (1.6)	1 (1.9)		<0.001
R	0	4 (0.3)	0	0	0		0.851
Total	1 (1.8)	4 (0.3)	0	2 (1.6)	1 (1.9)		0.047
M133						Immune escape	
I	0	2 (0.2)	1 (0.5)	1 (0.8)	0		0.576
L	2 (3.5)	22 (1.7)	16 (7.7)	3 (2.3)	0		<0.001
T	1 (1.8)	17 (1.3)	4 (1.9)	2 (1.6)	2 (3.8)		0.624
Total	3 (5.3)	41 (3.1)	21 (10.1)	6 (4.7)	2 (3.8)		<0.001
K141						Immune escape	
E	0	1 (0.1)	0	0	0		0.987
I	0	0	0	0	0		–
Total	0	1 (0.1)	0	0	0		0.987
P142						Immune escape	
L	0	0	0	0	0		–
S	0	1 (0.1)	0	0	0		0.987
Total	0	1 (0.1)	0	0	0		0.987
S/T143L	0	0	0	0	0	Immune escape	–
D144						Immune escape	
A	0	0	0	0	0		–
E	0	0	1 (0.5)	0	0		0.113
G	0	0	0	0	0		–
Total	0	0	1 (0.5)	0	0		0.113
G145						Immune escape	
A	0	3 (0.2)	1 (0.5)	0	0		0.889
R	0	3 (0.2)	1 (0.5)	0	0		0.889
Total	0	6 (0.5)	2 (1.0)	0	0		0.686
Escape mutants^*a*^	9 (15.8)	204 (15.6)	29 (14)	14 (10.9)	2 (3.8)	Immune escape	0.123
C1653T	2 (3.5)	14 (1.1)	1 (0.5)	1 (0.8)	10 (19.2)	HCC	<0.001
T1753V	10 (17.5)	45 (3.4)	10 (4.8)	2 (1.6)	11 (21.2)	HCC	<0.001
A1762T/G1764A	8 (14)	232 (17.7)	26 (12.6)	6 (4.7)	0	HCC	<0.001
Pre-S deletions	0	5 (0.4)	5 (2.4)	0	0	HCC	0.006
HCC-associated mutants^*b*^	11 (19.3)	250 (19.1)	34 (16.4)	7 (5.5)	16 (30.8)	HCC	<0.001
rtL180M	0	22 (1.7)	0	2 (1.6)	0	Antiviral resistance	0.252
rtA181						Antiviral resistance	
T	0	0	0	0	0		–
V	0	2 (0.2)	0	0	0		0.954
Total	0	2 (0.2)	0	0	0		0.954
rtT184						Antiviral resistance	
G	0	0	0	0	0		–
S	0	0	0	0	0		–
rtS202						Antiviral resistance	
G	0	2 (0.2)	1 (0.5)	0	0		0.807
I	0	0	0	0	0		–
Total	0	2 (0.2)	1 (0.5)	0	0		0.807
rtM204						Antiviral resistance	
I	0	17 (1.3)	0	24 (18.8)	0		<0.001
V	0	20 (1.5)	0	0	0		0.144
Total	0	37 (2.8)	0	24 (18.8)	0		<0.001
rtN236T	0	12 (0.9)	0	0	0	Antiviral resistance	0.393
rtM250V	0	0	0	0	0	Antiviral resistance	–
LAM-resistant mutants^*c*^	0	37 (2.8)	0	24 (18.8)	0	Antiviral resistance	<0.001
LdT-resistant mutants^*d*^	0	37 (2.8)	0	24 (18.8)	0	Antiviral resistance	<0.001
ADV-resistant mutants^*e*^	0	13 (1.0)	0	0	0	Antiviral resistance	0.350
ETV-resistant mutants^*f*^	0	0	0	0	0	Antiviral resistance	–
TDF/TAF-reduced susceptibility mutants^*g*^	0	1 (0.1)	0	0	0	Antiviral resistance	0.987

*The P-value was listed as “–” when the specific mutation was found in no genotype (statistical analysis not conducted). LAM, lamivudine; LdT, telbivudine; ADV, adefovir; ETV, entecavir; TDF, tenofovir disoproxil fumarate; TAF, tenofovir alafenamide; QS, quasi-subgenotype. ^a^Sequences with at least one immune escape mutation. ^b^Sequences with at least one HCC-associated mutation. ^c^Amino acid substitution profile for LAM resistance: rtM204V/I. ^d^Amino acid substitution profile for LdT resistance: rtM204I or L180M + M204V. ^e^Amino acid substitution profile for ADV resistance: rtA181T/V or rtN236T. ^f^Amino acid substitution profile for ETV resistance: rtL180M + rtM204I/V ± rtT184S/G ± rtS202I/G ± rtM250V. ^g^Amino acid substitution profile for TDF/TAF-reduced susceptibility: rtA181T/V + rtN236T.*

**TABLE 4:** Clinically relevant mutations in HBV/C subgenotypes.

	Subgenotypes, n (%)							
Sites	C1 (*n* = 451)	QS-C2 (*n* = 1518)	C3 (*n* = 28)	C4 (*n* = 13)	C5 (*n* = 19)	C6–C15 (*n* = 103)	Clinical issue	*P*
P120							Immune escape	
L	0	0	0	0	0	0		–
Q	0	0	0	0	0	0		–
S	0	2 (0.1)	0	0	0	0		0.976
T	1 (0.2)	7 (0.5)	0	0	0	0		0.945
Total	1 (0.2)	9 (0.6)	0	0	0	0		0.868
I/T126							Immune escape	
A	0	0	0	0	0	0		–
N	11 (2.4)	9 (0.6)	0	0	0	1 (1.0)		0.026
S	7 (1.6)	78 (5.1)	0	0	0	1 (1.0)		0.005
Total	18 (4.0)	87 (5.7)	0	0	0	2		0.166
Q129							Immune escape	
H	0	2 (0.1)	0	0	0	0		0.976
N	1 (0.2)	3 (0.2)	0	0	0	0		0.997
R	1 (0.2)	5 (0.3)	0	0	0	0		0.986
Total	2 (0.4)	10 (0.7)	0	0	0	0		0.936
G130							Immune escape	
N	1 (0.2)	24 (1.6)	0	0	0	0		0.177
R	1 (0.2)	4 (0.3)	0	0	0	0		0.994
Total	2 (0.4)	28 (1.8)	0	0	0	0		0.190
M133							Immune escape	
I	0	3 (0.2)	0	0	0	0		0.943
L	0	0	0	0	0	1 (1.0)		0.001
T	7 (1.6)	33 (2.2)	0	0	0	0		0.534
Total	7 (1.6)	36 (2.4)	0	0	0	1 (1.0)		0.674
K141							Immune escape	
E	0	1 (0.1)	0	0	0	0		0.995
I	0	2 (0.1)	0	0	0	0		0.976
Total	0	3 (0.2)	0	0	0	0		0.943
P142							Immune escape	
L	1 (0.2)	0	0	0	0	0		0.589
S	0	0	0	0	0	0		–
Total	1 (0.2)	0	0	0	0	0		0.589
S/T143L	1 (0.2)	0	0	0	0	0	Immune escape	0.589
D144							Immune escape	
A	1 (0.2)	0	0	0	0	0		0.589
E	1 (0.2)	0	1 (3.6)	0	0	0		<0.001
G	0	1 (0.1)	0	0	0	0		0.995
Total	2 (0.4)	1 (0.1)	1 (3.6)	0	0	0		0.001
G145							Immune escape	
A	3 (0.7)	22 (1.4)	1 (3.6)	0	0	0		0.445
R	4 (0.9)	7 (0.5)	0	0	0	35 (34)		<0.001
Total	7 (1.6)	29 (1.9)	1 (3.6)	0	0	35 (34)		<0.001
Escape mutants^*a*^	37 (8.2)	165 (10.9)	2 (7.1)	0	0	38 (36.9)	Immune escape	<0.001
C1653T	36 (8.0)	205 (13.5)	1 (3.6)	0	0	15 (14.6)	HCC	0.005
T1753V	99 (22)	262 (17.3)	2 (7.1)	1 (7.7)	0	19 (18.4)	HCC	0.026
A1762T/G1764A	185 (41)	725 (47.8)	6 (21.4)	2 (15.4)	3 (15.8)	58 (56.3)	HCC	<0.001
Pre-S deletions	10 (2.2)	56 (3.7)	3 (10.7)	1 (7.7)	0	1 (1.0)	HCC	0.067
HCC-associated mutants^*b*^	201 (44.6)	782 (51.5)	7 (25)	3 (23.1)	3 (15.8)	59 (57.3)	HCC	<0.001
rtL180M	10 (2.2)	75 (4.9)	0	0	2 (10.5)	1 (1.0)	Antiviral resistance	0.023
rtA181							Antiviral resistance	
T	1 (0.2)	2 (0.1)	0	0	0	0		0.994
V	0	15 (1.0)	0	0	0	0		0.296
Total	1 (0.2)	17 (1.1)	0	0	0	0		0.434
rtT184							Antiviral resistance	
G	0	0	0	0	0	0		–
S	0	0	0	0	0	1 (1.0)		0.001
Total	0	0	0	0	0	1 (1.0)		0.001
rtS202							Antiviral resistance	
G	1 (0.2)	4 (0.3)	0	0	0	0		0.994
I	0	0	0	0	0	0		–
Total	1 (0.2)	4 (0.3)	0	0	0	0		0.994
rtM204							Antiviral resistance	
I	4 (0.9)	121 (8.0)	0	0	0	0		<0.001
V	7 (1.6)	50 (3.3)	0	0	2 (10.5)	1 (1.0)		0.062
Total	11 (2.4)	171 (11.3)	0	0	2 (10.5)	1 (1.0)		<0.001
rtN236T	0	4 (0.3)	0	0	0	0	Antiviral resistance	0.899
rtM250V	0	3 (0.2)	0	0	0	0	Antiviral resistance	0.943
LAM-resistant mutants^*c*^	11 (2.4)	171 (11.3)	0	0	2 (10.5)	1 (1.0)	Antiviral resistance	<0.001
LdT-resistant mutants^*d*^	10 (2.2)	168 (11.1)	0	0	2 (10.5)	1 (1.0)	Antiviral resistance	<0.001
ADV-resistant mutants^*e*^	1 (0.2)	19 (1.3)	0	0	0	0	Antiviral resistance	0.343
ETV-resistant mutants^*f*^	0	6 (0.4)	0	0	0	1 (1.0)	Antiviral resistance	0.671
TDF/TAF-reduced susceptibility mutants^*g*^	0	2 (0.1)	0	0	0	0	Antiviral resistance	0.976

*The P-value was listed as “–” when the specific mutation was found in no genotype (statistical analysis not conducted). LAM, lamivudine; LdT, telbivudine; ADV, adefovir; ETV, entecavir; TDF, tenofovir disoproxil fumarate; TAF, tenofovir alafenamide; QS, quasi-subgenotype. ^a^Sequences with at least one immune escape mutation. ^b^Sequences with at least one HCC-associated mutation. ^c^Amino acid substitution profile for LAM resistance: rtM204V/I. ^d^Amino acid substitution profile for LdT resistance: rtM204I or L180M + M204V. ^e^Amino acid substitution profile for ADV resistance: rtA181T/V or rtN236T. ^f^Amino acid substitution profile for ETV resistance: rtL180M + rtM204I/V ± rtT184S/G ± rtS202I/G ± rtM250V. ^g^Amino acid substitution profile for TDF/TAF-reduced susceptibility: rtA181T/V + rtN236T.*

**TABLE 5:** Clinically relevant mutations in HBV/D subgenotypes.

	Subgenotypes, n (%)							
Sites	D1 (*n* = 516)	D2 (*n* = 291)	D3 (*n* = 189)	D4 (*n* = 21)	D5 (*n* = 21)	D6 (*n* = 7)	Clinical issue	*P*
P120							Immune escape	
L	0	0	1 (0.5)	0	0	0		0.475
Q	1 (0.2)	0	0	0	0	0		0.960
S	13 (2.5)	0	1 (0.5)	0	0	0		0.052
T	2 (0.4)	1 (0.3)	0	4 (19)	1 (4.8)	0		<0.001
Total	16 (3.1)	1 (0.3)	2 (1.1)	4 (19)	1 (4.8)	0		<0.001
I/T126							Immune escape	
A	0	0	0	0	0	0		–
N	1 (0.2)	1 (0.3)	0	0	0	0		0.976
S	2 (0.4)	0	0	0	0	0		0.842
Total	3 (0.6)	1 (0.3)	0	0	0	0		0.918
Q129							Immune escape	
H	2 (0.4)	0	0	0	0	0		0.006
N	0	0	0	0	0	0		–
R	9 (1.7)	0	0	4 (19)	1 (4.8)	0		<0.001
Total	11 (2.1)	0	0	4 (19)	1 (4.8)	0		<0.001
G130							Immune escape	
N	3 (0.6)	0	0	0	0	0		0.687
R	0	0	0	0	0	0		–
Total	3 (0.6)	0	0	0	0	0		0.687
M133							Immune escape	
I	2 (0.4)	1 (0.3)	0	0	0	0		0.970
L	0	1 (0.3)	0	0	0	0		0.762
T	1 (0.2)	0	0	0	0	0		0.960
Total	3 (0.6)	2 (0.7)	0	0	0	0		0.910
K141							Immune escape	
E	0	0	0	0	0	0		–
I	0	0	0	0	0	0		–
P142							Immune escape	
L	1 (0.2)	1 (0.3)	0	0	0	0		0.976
S	0	0	0	0	0	0		–
Total	1 (0.2)	1 (0.3)	0	0	0	0		0.976
S/T143L	13 (2.5)	0	6 (3.2)	0	0	0	Immune escape	0.085
D144							Immune escape	
A	0	0	0	0	0	0		–
E	2 (0.4)	2 (0.7)	0	0	0	0		0.899
G	1 (0.2)	0	0	0	0	0		0.960
Total	3 (0.6)	2 (0.7)	0	0	0	0		0.910
G145							Immune escape	
A	2 (0.4)	0	0	0	0	0		0.842
R	1 (0.2)	24 (8.2)	1 (0.5)	0	0	0		<0.001
Total	3 (0.6)	24 (8.2)	1 (0.5)	0	0	0		<0.001
Escape mutants^*a*^	51 (9.9)	30 (10.3)	9 (4.8)	4 (19)	2 (9.5)	0	Immune escape	0.139
C1653T	31 (6.0)	38 (13.1)	18 (9.5)	1 (4.8)	2 (9.5)	0	HCC	0.023
T1753V	118 (22.9)	36 (12.4)	14 (7.4)	0	0	1 (14.3)	HCC	<0.001
A1762T/G1764A	146 (28.3)	42 (14.4)	29 (15.3)	3 (14.3)	4 (19)	1 (14.3)	HCC	<0.001
Pre-S deletions	0	0	0	0	0	0	HCC	–
HCC-associated mutants^*b*^	188 (36.4)	82 (28.2)	41 (21.7)	3 (14.3)	4 (19)	1 (14.3)	HCC	0.001
rtL180M	12 (2.3)	48 (16.5)	0	0	0	0	Antiviral resistance	<0.001
rtA181							Antiviral resistance	
T	0	0	0	0	0	0		–
V	1 (0.2)	0	0	0	0	0		0.960
Total	1 (0.2)	0	0	0	0	0		0.960
rtT184							Antiviral resistance	
G	0	0	0	0	0	0		–
S	1 (0.2)	0	0	0	0	0		0.960
Total	1 (0.2)	0	0	0	0	0		0.960
rtS202							Antiviral resistance	
G	0	1 (0.3)	0	0	0	0		0.762
I	0	1 (0.3)	0	0	0	0		0.762
Total	0	2 (0.7)	0	0	0	0		0.393
rtM204							Antiviral resistance	
I	10 (1.9)	25 (8.6)	14 (7.4)	0	1 (4.8)	0		<0.001
V	5 (1.0)	22 (7.6)	0	0	0	0		<0.001
Total	15 (2.9)	47 (16.2)	14 (7.4)	0	1 (4.8)	0		<0.001
rtN236T	0	0	0	0	0	0	Antiviral resistance	–
rtM250V	0	0	0	0	0	0	Antiviral resistance	–
LAM-resistant mutants^*c*^	15 (2.9)	47 (16.2)	14 (7.4)	0	1 (4.8)	0	Antiviral resistance	<0.001
LdT-resistant mutants^*d*^	15 (2.9)	47 (16.2)	14 (7.4)	0	1 (4.8)	0	Antiviral resistance	<0.001
ADV-resistant mutants^*e*^	1 (0.2)	0	0	0	0	0	Antiviral resistance	0.960
ETV-resistant mutants^*f*^	1 (0.2)	1 (0.3)	0	0	0	0	Antiviral resistance	0.976
TDF/TAF-reduced susceptibility mutants^*g*^	0	0	0	0	0	0	Antiviral resistance	–

*The P-value was listed as “–” when the specific mutation was found in no genotype (statistical analysis not conducted). LAM, lamivudine; LdT, telbivudine; ADV, adefovir; ETV, entecavir; TDF, tenofovir disoproxil fumarate; TAF, tenofovir alafenamide. ^a^Sequences with at least one immune escape mutation. ^b^Sequences with at least one HCC-associated mutation. ^c^Amino acid substitution profile for LAM resistance: rtM204V/I. ^d^Amino acid substitution profile for LdT resistance: rtM204I or L180M + M204V. ^e^Amino acid substitution profile for ADV resistance: rtA181T/V or rtN236T. ^f^Amino acid substitution profile for ETV resistance: rtL180M + rtM204I/V ± rtT184S/G ± rtS202I/G ± rtM250V. ^g^Amino acid substitution profile for TDF/TAF-reduced susceptibility: rtA181T/V + rtN236T.*

**TABLE 6:** Clinically relevant mutations in HBV/F subgenotypes.

	Subgenotypes, n (%)					
Sites	F1 (*n* = 123)	F2 (*n* = 29)	F3 (*n* = 39)	F4 (*n* = 56)	Clinical issue	*P*
P120					Immune Escape	
L	0	0	0	0		–
Q	0	0	0	1 (1.8)		0.331
S	0	0	0	0		–
T	0	0	0	0		–
Total	0	0	0	1 (1.8)		0.331
I/T126					Immune escape	
A	0	1 (3.4)	1 (2.6)	0		0.140
N	0	0	0	0		–
S	0	0	0	0		–
Total	0	1 (3.4)	1 (2.6)	0		0.140
Q129					Immune escape	
H	0	0	0	0		–
N	0	0	0	0		–
R	0	0	0	0		–
G130					Immune escape	
N	0	0	0	0		–
R	0	1 (3.4)	0	0		0.056
Total	0	1 (3.4)	0	0		0.056
M133					Immune escape	
I	0	0	0	0		–
L	0	0	0	0		–
T	0	0	0	0		–
K141					Immune escape	
E	0	0	0	0		–
I	0	0	0	0		–
P142					Immune escape	
L	0	0	0	0		–
S	0	0	0	0		–
S/T143L	0	0	0	0	Immune escape	–
D144					Immune escape	
A	0	0	0	0		–
E	1 (0.8)	0	0	0		0.798
G	0	0	0	0		–
Total	1 (0.8)	0	0	0		0.798
G145					Immune escape	
A	0	0	0	0		–
R	0	0	0	0		–
Escape mutants^*a*^	1 (0.8)	2 (6.9)	1 (2.6)	1 (1.8)	Immune escape	0.216
C1653T	3 (2.4)	2 (6.9)	0	1 (1.8)	HCC	0.319
T1753V	6 (4.9)	13 (44.8)	4 (10.3)	9 (16.1)	HCC	<0.001
A1762T/G1764A	36 (29.3)	8 (27.6)	6 (15.4)	6 (10.7)	HCC	0.027
Pre-S deletions	3 (2.4)	0	0	0	HCC	0.382
HCC-associated mutants^*b*^	38 (30.9)	15 (51.7)	7 (17.9)	15 (26.8)	HCC	0.024
rtL180M	5 (4.1)	2 (6.9)	0	0	Antiviral resistance	0.159
rtA181					Antiviral resistance	
T	0	0	0	0		–
V	0	0	0	0		–
rtT184					Antiviral resistance	
G	0	0	0	0		–
S	0	0	0	0		–
rtS202					Antiviral resistance	
G	0	0	0	0		–
I	0	0	0	0		–
rtM204					Antiviral resistance	
I	0	0	0	0		–
V	4 (3.3)	2 (6.9)	0	0		0.161
Total	4 (3.3)	2 (6.9)	0	0		0.161
rtN236T	0	0	0	0	Antiviral resistance	–
rtM250V	0	0	0	0	Antiviral resistance	–
LAM-resistant mutants^*c*^	4 (3.3)	2 (6.9)	0	0	Antiviral resistance	0.161
LdT-resistant mutants^*d*^	4 (3.3)	2 (6.9)	0	0	Antiviral resistance	0.161
ADV-resistant mutants^*e*^	0	0	0	0	Antiviral resistance	–
ETV-resistant mutants^*f*^	0	0	0	0	Antiviral resistance	–
TDF/TAF-reduced susceptibility mutants^*g*^	0	0	0	0	Antiviral resistance	–

*The P-value was listed as “–” when the specific mutation was found in no genotype (statistical analysis not conducted). LAM, lamivudine; LdT, telbivudine; ADV, adefovir; ETV, entecavir; TDF, tenofovir disoproxil fumarate; TAF, tenofovir alafenamide. ^a^Sequences with at least one immune escape mutation. ^b^Sequences with at least one HCC-associated mutation. ^c^Amino acid substitution profile for LAM resistance: rtM204V/I. ^d^Amino acid substitution profile for LdT resistance: rtM204I or L180M + M204V. ^e^Amino acid substitution profile for ADV resistance: rtA181T/V or rtN236T. ^f^Amino acid substitution profile for ETV resistance: rtL180M + rtM204I/V ± rtT184S/G ± rtS202I/G ± rtM250V. ^g^Amino acid substitution profile for TDF/TAF-reduced susceptibility: rtA181T/V + rtN236T.*

### HCC-Associated Mutations

The frequencies of C1653T, T1753V and A1762T/G1764A (double mutant) and Pre-S deletions in different HBV genotypes are shown in Table 1. HCC-associated mutations were detected in 2,183 out of 6,479 sequences (33.7%). HBV genotype G showed the highest frequency of mutations (97.5%), followed by genotype C (49.7%), D (30.9%), F (30.5%), A (29.9%), E (24.3%), B (18.1%), and H (15.4%) (*P* < 0.001). C1653T, T1753V and A1762T/G1764A were highly frequent in genotype G (95, 95, and 97.5%, respectively), followed by genotype C (12, 18.1, and 46.1%, respectively), while Pre-S deletions prevailed in genotype C (3.3%) (Table 1).

All HBV subgenotypes displayed HCC-associated gene variations. QS-A3, B5, C6–C15, D1 and F2 displayed the highest mutation frequencies within their respective genotypes (QS-A3: 35.4%; B5: 30.8%; C6–C15: 57.3%; D1: 36.4%; F2: 51.7%). Significant differences in A1762T/G1764A rates were found within genotypes B, C, D, and F, with subgenotypes B2, C6-C15, D1 and F1 showing the highest rates (17.7, 56.3, 28.3, and 29.3%, respectively). Similarly, Pre-S deletion frequencies were markedly different within genotypes A and B, with the highest rates detected for subgenotypes A1 and QS-B3 (3.0 and 2.4%, respectively, *P* < 0.05 for both) (Tables 2–6).

### Antiviral Resistance Mutations

Seven amino acid positions within the RT domain of HBV polymerase involving 11 mutations associated with resistance to NAs were analyzed. The frequencies of mutations and resistant mutants for each NA in different HBV genotypes are shown in Table 1. Overall, rtL180M, rtA181T/V, rtT184G/S, rtS202G/I, rtM204I, rtM204V, rtN236T, and rtM250V were more frequently detected in genotypes A (13.9%), C (0.8%), D (0.1%), H (3.8%), G (25%), A (13.1%), B (0.7%), and C (0.1%), respectively. Significant differences were evident among HBV genotypes in terms of resistance to NAs. LAM- and LdT-resistant mutants were more frequent in genotype G (32.5% for both), followed by genotypes A (14.1 and 13.8%), C (8.6 and 8.4%), H (7.7% for both), D (7.4% for both), B (3.5% for both), F (3.2% for both) and E (0.3% for both) (*P* < 0.001). ADV-resistant mutants were only found in genotypes C (0.9%), B (0.7%), A (0.1%), and D (0.1%) (*P* = 0.016), whereas ETV-resistant mutants were specifically detected in genotypes H (3.8%), C (0.4%), and D (0.2%) (*P* < 0.001). Only genotypes B and C contained mutations with reduced susceptibility to TDF and TAF (0.1% for both genotypes) (Table 1).

The HBV subgenotypes A2, B4, QS-C2, D2 and F2 displayed the highest frequencies of LAM- and LdT-resistant mutants within their respective genotypes (A2: 21.5 and 21.3%; B4: 18.8% for both; QS-C2: 11.3% and 11.1%; D2: 16.2% for both; F2: 6.9% for both). Subgenotypes A2, B2, QS-C2 and D1 showed the highest frequencies of ADV-resistant mutants within their respective genotypes (A2: 0.2%; B2: 1.0%; QS-C2: 1.3%; D1: 0.2%). Subgenotypes C6–C15 and D2 showed the highest frequencies of ETV-resistant mutants within their respective genotypes (C6–C12: 1.0%; D2: 0.3%). Only B2 and QS-C2 had mutants with reduced susceptibility to TDF and TAF (0.1% for both) (Tables 2–6).

### Geographic Distribution of HBV Mutants

**FIGURE 3:**
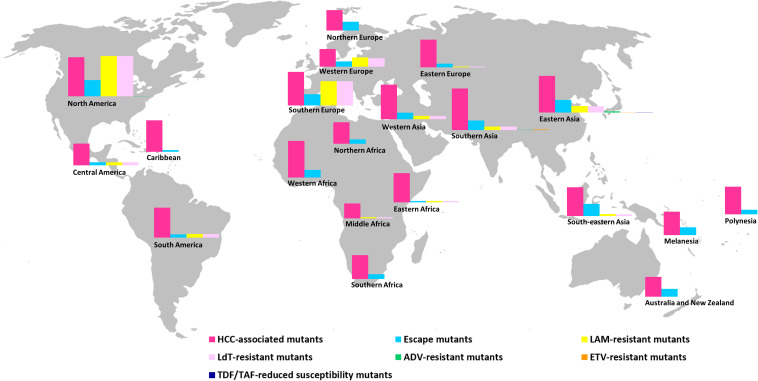
Prevalence of HBV mutants in different geographic regions based on 6,207 complete genome sequences present in the HBVdb database. The map was reconstructed using Wikimedia Commons (https://commons.wikimedia.org/wiki/File:BlankMap-World-noborders.png), this figure is similar but not identical to the original image and is therefore for illustrative purposes only.

The frequencies of HBV mutants according to geographic region are shown in Figure 3 and Table 7. Central Asia was the only region where no mutants were detected. The highest rates of HCC-associated mutants were observed in Southern Asia (41%), followed by North America (37%) and Eastern Asia (36.5%) and the lowest rates in Middle Africa (14.6%), Western Europe (17.5%), and Australia and New Zealand (19.6%) (*P* < 0.001). Immune escape mutants were detected more often in North America (16.3%), Eastern Asia (12.3%) and South-eastern Asia (12%) and less frequently in Middle Africa (0), Eastern Africa (1.4%) and the Caribbean (1.6%) (*P* < 0.001). Regarding antiviral therapy resistance, HBV sequences from Australia and New Zealand, Caribbean, Melanesia, Northern Africa, Northern Europe, Polynesia, Southern Africa, and Western Africa contained no NA-resistant mutations. Higher frequencies of LAM- and LdT-resistant mutants were found in North America (40.9% for both), Southern Europe (24% for both) and Western Europe (9.1 and 8.4%), and lower frequencies in Eastern Europe (0.7% for both), Middle Africa (1.0% for both) and Eastern Africa (1.4% for both) (*P* < 0.001). ADV-resistant mutants were specifically identified in Eastern Asia (1.1%), North America (0.2%) and Southern Asia (0.2%) and ETV-resistant mutants in Southern Asia (0.4%) and Eastern Asia (0.1%) (*P* > 0.05 for all). Mutants with reduced TDF and TAF susceptibility were solely detected in Eastern Asia (0.1%) (Figure 3 and Table 7).

**TABLE 7:** Clinically relevant HBV mutants by geographic region.

	Geographic Regions, n (%)																					
Clinical issue	Australia and New Zealand (n = 51)	Caribbean (n = 61)	Central America (n = 70)	Central Asia (n = 13)	Eastern Africa (n = 69)	Eastern Asia (n = 3118)	Eastern Europe (n = 139)	Melanesia (n = 13)	Middle Africa (n = 96)	Northern Africa (n = 47)	North America (n = 443)	Northern Europe (n = 35)	Polynesia (n = 22)	South America (n = 342)	South-eastern Asia (n = 616)	Southern Africa (n = 106)	Southern Asia (n = 458)	Southern Europe (n = 54)	Western Africa (n = 174)	Western Asia (n = 126)	Western Europe (n = 154)	P
LAM-resistant mutants^*a*^	0	0	2 (2.9)	0	1 (1.4)	199 (6.4)	1 (0.7)	0	1 (1.0)	0	181 (40.9)	0	0	12 (3.5)	12 (1.9)	0	15 (3.3)	13 (24)	0	4 (3.2)	14 (9.1)	< 0.001
LdT-resistant mutants^*b*^	0	0	2 (2.9)	0	1 (1.4)	196 (6.3)	1 (0.7)	0	1 (1.0)	0	181 (40.9)	0	0	12 (3.5)	11 (1.8)	0	15 (3.3)	13 (24)	0	4 (3.2)	13 (8.4)	< 0.001
ADV-resistant mutants^*c*^	0	0	0	0	0	33 (1.1)	0	0	0	0	1 (0.2)	0	0	0	0	0	1 (0.2)	0	0	0	0	0. 112
ETV-resistant mutants^*d*^	0	0	0	0	0	8 (0.3)	0	0	0	0	0	0	0	0	0	0	2 (0.4)	0	0	0	0	0. 991
TDF/TAF-reduced susceptibility mutants^*e*^	0	0	0	0	0	3 (0.1)	0	0	0	0	0	0	0	0	0	0	0	0	0	0	0	1.000
Escape mutants^*f*^	4 (7.8)	1 (1.6)	2 (2.9)	0	1 (1.4)	385 (12.3)	5 (3.6)	1 (7.7)	0	2 (4.3)	72 (16.3)	3 (8.6)	1 (4.5)	11 (3.2)	74 (12)	5 (4.7)	43 (9.4)	6 (11.1)	13 (7.5)	8 (6.3)	8 (5.2)	< 0.001
HCC-associated mutants^*g*^	10 (19.6)	19 (31.1)	15 (21.4)	0	20 (29)	1139 (36.5)	38 (27.3)	3 (23.1)	14 (14.6)	10 (21.3)	164 (37)	7 (20)	6 (27.3)	101 (29.5)	175 (28.4)	25 (23.6)	188 (41)	18 (33.3)	63 (36.2)	43 (34.1)	27 (17.5)	<0.001

*LAM. lamivudine; LdT. telbivudine; ADV. adefovir; ETV. entecavir; TDF. tenofovir disoproxil fumarate; TAF. tenofovir alafenamide. ^a^Amino acid substitution profile for LAM resistance: rtM204V/I. ^b^Amino acid substitution profile for LdT resistance: rtM204I or L180M + M204V. ^c^Amino acid substitution profile for ADV resistance: rtA181T/V or rtN236T. ^d^Amino acid substitution profile for ETV resistance: rtL180M + rtM204I/V ± rtT184S/G ± rtS202I/G ± rtM250V. ^e^Amino acid substitution profile for TDF/TAF-reduced susceptibility: rtA181T/V + rtN236T. ^f^Sequences with at least one immune escape mutation. ^g^Sequences with at least one HCC-associated mutation.*

The comparison of mutation profiles of same (sub)genotype among geographic regions is shown in Supplementary Table S2. A1 isolates from Southern Asia displayed significantly higher rates of HCC-associated mutants (61.4%) than A1 isolates from Caribbean (32.1%), Eastern Africa (25%), South America (24%), and Southern Africa (21.9%) (*P* < 0.05). Similar results were observed between C isolates from Asia (Southern Asia, 57.1%; Eastern Asia, 50.5%; South-eastern Asia, 43.4%) and Oceania (Australia and New Zealand, 20%; Melanesia, 25%; Polynesia, 27.3%) (*P* < 0.05), E isolates from Western Africa and Middle Africa (33.1% and 8.5%, respectively, *P* < 0.05), and F isolates from North America and South America (87.5% and 30%, respectively, *P* < 0.05). A2 isolates from North America and Western Europe showed significant differences in the rates of HCC-associated mutants (40.3 and 9.3%, respectively, *P* < 0.05) and immune escape mutants (16.5 and 3.7%, respectively, *P* < 0.05). Moreover, isolates from North America showed the highest frequencies of LAM-resistant mutants within (sub)genotypes A2, B, D, and G (*P* < 0.05) (Supplementary Table S2).

## Discussion

Evolution is an important aspect of epidemiology of viral diseases. HBV evolution occurs through both mutation and recombination processes (Kay and Zoulim, 2007; Araujo, 2015). Since HBV replication involves an error-prone reverse transcription step, nucleotide substitution rates are higher than those for other DNA viruses. The estimated HBV mutation rates are reported to range from 10^–4^ to 10^–6^ substitutions/site/year (Zehender et al., 2014; Paraskevis et al., 2015; Spitz et al., 2019). This high substitution rate allows HBV to evolve rapidly and adapt to changes in the host environment, leading to the emergence of mutations with important implications for prevention, diagnosis, treatment, and prognosis of infection. HBV mutants have been documented worldwide (Zhang et al., 2016), indicating the potential to spread in a range of human populations and develop their own epidemiology.

HBV genotypes and subgenotypes have been associated with differences in disease progression, response to antiviral therapy, and clinical outcomes (Kramvis and Kew, 2005; McMahon, 2009; Kim et al., 2011; Liu and Kao, 2013). One potential reason is that HBV mutations may be more common among some (sub)genotypes than among others. However, due to the unique distribution patterns of HBV genotypes in Asian and Western countries, epidemiological data on HBV mutants are based on comparisons between genotypes B and C or A and D, with little information on the other genotypes. In this study, mutations related to immune escape, antiviral resistance, and HCC development were analyzed in 6,479 HBV genome sequences classified into genotypes A–H. To our knowledge, the present study has conducted the most comprehensive analysis of the association of clinically relevant mutations of HBV with genotypes, subgenotypes, and geographic regions.

Overall, immune escape mutations were detected in 10.7% HBV genomes. Similar rates have been determined in cohorts from Argentina (7.5–10.7%) (Di Lello et al., 2019), China (9.01%) (Yan et al., 2017), Spain (6.6–12.5%) (Avellon and Echevarria, 2006), and Turkey (8.3%) (Sayan et al., 2010). Only 4 of the 25 escape mutations had total frequencies of ≥1% (I/T126S, 1.8%; G145R, 1.2%; M133T, 1.2%; and Q129R, 1.0%), consistent with a previous large-scale analysis showing frequencies of no less than 1% for most escape mutations (Ma and Wang, 2012). Particularly, the most common escape mutation identified, I/T126S, was previously associated with vaccine failure in Chinese adults undergoing the HBV vaccination program (He et al., 2001) and most prevalent among patients under 15 years of age after mass vaccination in China (Yan et al., 2017).

Notably, significant differences in the frequencies of escape mutations among HBV genotypes were observed in this study, with genotypes A–D and G showing higher rates than E, F, and H. The lack of escape mutations observed in genotype H should be interpreted with caution due to the low number of genotype H sequences available for analysis. However, similar findings were reported by Ma and Wang (2012), who showed that HBV genotypes A-D harbor more escape mutations than the other genotypes. Likewise, Di Lello et al. (2019) observed a significantly higher rate of escape mutations in genotype D (33%) than genotype F (2.3%). The results collectively suggest low circulation of immune escape mutants among the Amerindian (and more divergent) HBV genotypes F and H, and highlight the necessity to monitor genotypes A (mainly A2), B, C (mainly C6-C15), D, and G.

Moreover, since the HBV vaccine used worldwide is produced with HBsAg subgenotype A2, stronger selection pressure of the humoral immune response against the “a” determinant of HBV-A2 isolates may occur. Consequently, higher rates of immune escape mutants are expected in subgenotype A2 than other (sub)genotypes. In fact, significant differences in the frequencies of immune escape mutants were observed within genotype A, with A2 showing the highest rates. However, other (sub)genotypes included higher rates of escape mutants than A2, suggesting that this potential selective pressure on subgenotype A2 is not yet a concern for virus vaccine design.

Hepatocellular carcinoma development due to HBV infection is a multifactorial process associated with viral, host and environmental factors. Patients with chronic HBV infection have a 20-fold higher risk of HCC than non-infected individuals (Parkin, 2006). The ability to identify patients at risk of HCC is crucial, since progression to liver cancer in the absence of cirrhosis has been documented in chronic HBV infection cases (McMahon, 2009; Chayanupatkul et al., 2017; Rajoriya et al., 2017).

A previous meta-analysis showed that Pre-S deletions, C1653T in enhancer II, and T1753V and A1762T/G1764A in BCP are associated with increased risk of HCC compared with HBV without mutations (Liu et al., 2009). Significant differences in these mutations were observed among HBV genotypes (*P* < 0.001). Genotype G showed by far the highest frequency (≥95%) of C1653T, T1753V, and A1762T/G1764A. Genotype G is associated with more severe liver disease in HIV-HBV co-infected patients (Lacombe et al., 2006; Dao et al., 2011; Malagnino et al., 2019) but its role in HCC development remains to be established. Genotype C displayed the highest frequency of Pre-S deletions (3.3%) and the second highest rates of C1653T, T1753V and A1762T/G1764A variations. These findings are consistent with several previous reports demonstrating the association of genotype C with increased risk of HCC relative to other major HBV genotypes (Chan et al., 2004; Liu et al., 2009; Wong et al., 2013).

Interestingly, a correlation between genotype F infection and development of HCC among native Alaskan people was demonstrated in an earlier study (Livingston et al., 2007). Furthermore, Kowalec et al. (2013) showed that development of HCC in the native Alaskan population is significantly associated with specific mutations within the core promoter region of genotype F isolates (Kowalec et al., 2013). In the current study, all genotype F sequences from Alaska were classified as subgenotype F1. On the other hand, subgenotype F2 has been associated with development of HCC in Venezuelan patients (Puche et al., 2016; Pujol et al., 2020). In fact, both subgenotypes showed similar frequencies of A1762T/G1764A (F1, 29.3%; F2, 27.6%), which were higher than those observed for subgenotypes F3 and F4 (15.4% and 10.7%, *P* = 0.027). Moreover, significantly higher frequency of T1753V was observed in subgenotype F2 (44.8%, *P* < 0.001), whereas F1 was the only subgenotype that contained Pre-S deletions. The collective results suggest that HBV subgenotypes F1 and F2 are more prone to harboring HCC-associated mutations than subgenotypes F3 and F4, resulting in stronger oncogenic potential.

Likewise, the frequency of Pre-S deletions in subgenotype A1 was significantly higher than that in other A subgenotypes (3.0%, *P* = 0.008) and slightly lower than that in genotype C (3.3%). Notably, 62.5% (5 of 8) of A1 sequences with Pre-S deletions were obtained from African patients (Supplementary Table S1). The high frequency of Pre-S deletions in A1 may contribute to the enhanced pathogenicity of this subgenotype and its association with high rates of HCC in sub-Saharan Africa (Kew et al., 2005; Kramvis and Kew, 2007). In contrast, the predominance of genotype H among the Mexican population is associated with low prevalence of HCC (Roman and Panduro, 2013). Consistently, genotype H showed the lowest rates of T1753V and A1762T/G1764A and no Pre-S deletions, and was identified as the HBV genotype with fewer HCC-associated mutant sequences.

Although NA therapies have been successful in sustained viral suppression, long-term use of treatments with a low barrier to resistance contributes to the emergence of resistant HBV mutants. Therefore, such agents (LAM, LdT and ADV) are no longer recommended as first-line therapy (Easl, 2017; Terrault et al., 2018). However, management of NA failure remains a crucial issue, especially in low-resource countries where ETV, TDF, and TAF are not available for naïve patients or treatment-experienced patients. Consistently, the overall rates of mutations inducing resistance to LAM and LdT were higher than those for other NAs (LAM, 7.3%; LdT, 7.2%; ADV, 0.5%; ETV, 0.2%). In addition, very low rates of mutants with reduced susceptibility to TDF and TAF (0.05%, 3/6479) were observed.

The link between genotype and response to treatment with NA-based regimens is still unclear. Results from the current study showed significant differences in the rates of LAM-, LdT-, ADV-, and ETV-resistant mutants among HBV genotypes and subgenotypes. Genotype G showed the highest rates of LAM- and LdT-resistant mutants, whereas genotype E had the lowest ones (32.5 and 0.3% for both NAs, respectively, *P* < 0.001). However, these differences may not be exclusively due to intrinsic characteristics of HBV genotypes. Notably, genotype G has been frequently found in HIV-infected men who have sex with men (MSM) (Sanchez et al., 2007; Osiowy et al., 2008; Araujo et al., 2013; Cornelissen et al., 2016), suggesting a strong association with this risk group. In addition, LAM has been widely used in the treatment of both HBV and HIV viruses. This may lead to a stronger selective pressure due to the more frequent use of LAM on genotype G isolates than other genotypes, ultimately resulting in higher frequencies of LAM-resistant mutants (and LdT due to cross-resistance). Likewise, genotype E is highly endemic in most sub-Saharan Africa (Andernach et al., 2013) where therapy is not always accessible to HBV chronic patients (Spearman et al., 2017). Consequently, weaker antiviral selection pressure on HBV isolates may occur in this region than in others where NA-based regimens are widely available, restricting the expansion of genotype E resistant mutants throughout populations.

Interestingly, significant differences in mutation profiles of same (sub)genotype among geographic regions were observed. In this regard, the differences in the rates of HCC-associated mutants between genotype C isolates from Asia and Oceania, and genotype F isolates from North America and South America, may be due to the circulation of distinct subgenotypes in these regions. On the other hand, subgenotype A2 isolates from North America showed significantly higher rates of immune escape and HCC-associated mutants than those from Western Europe. Likewise, genotype E isolates from Western Africa had significantly more HCC mutations than those from Middle Africa. Moreover, the highest rates of LAM-resistant mutants within (sub)genotypes A2, B, D, and G were observed in North America. Altogether, these findings suggest that the same (sub)genotype may have different potentials for immune escape, oncogenicity and treatment response, depending on the selective pressures (e.g., host genetic background, prophylactic and therapeutic regimens) applied in the geographic locations.

According to Carman’s study (Carman, 1997), viral variants occur naturally and may have been selected over centuries, while viral mutants have been selected over a short period by human intervention (therapy or prophylaxis). It is not always clear that a sequence substitution is a functional mutation (mutant) or simply a characteristic (variant) of a specific (sub)genotype. Variants are expected to be more frequent than mutants within (sub)genotypes. Interestingly, the antiviral resistance- and immune escape mutations analyzed in our study showed much lower frequencies within (sub)genotypes than HCC-associated mutations. This might be due to the fact that therapy and prophylaxis are recent selective forces, while HCC-associated mutations have been selected during much longer time by host immune pressure. Here, the vast majority of the mutations showed low frequencies within (sub)genotypes, and therefore, do not seem to be variants of any specific group. However, we have demonstrated that some mutations were biased by (sub)genotype. It is possible that over the years of viral evolution, they will become variants within these groups. This have occurred with BCP mutations in genotype G (frequency rate ≥ 95%), which have been stably integrated into the genome. The analysis of such a large dataset provided in this study will be helpful for further studies in establishing the likelihood that a sequence substitution is actually a mutant or a variant of a specific HBV (sub)genotype.

Finally, a number of limitations of this study need to be considered. Our analyses were based on HBV sequences from public databases, which may not be entirely representative of the globally circulating strains. Additionally, each sequence was considered a single HBV isolate, not taking into account the occurrence of serial sequences representing the same single isolate (e.g., clones), which may have generated some bias in our results. However, the large number of sequences analyzed may have overcome these limitations, at least in part. Moreover, information on whether the sequence was obtained before or after antiviral treatment or HCC development was not obtained here, which would have contributed to an accurate analysis for the relationship between mutation and clinical outcome. Nevertheless, since obtaining such information from all sequences was not possible, only mutations that have been strongly associated with immune escape, antiviral resistance, and HCC development in previous studies were selected for analysis. Lastly, due to the large variation in the number of sequences available for each (sub)genotype, over- or underrepresentation of some (sub)genotypes may have influenced the results. Therefore, although some results corroborated previous data, others should be further confirmed.

## Conclusion

We have comprehensively investigated HBV mutations with clinical implications and their associations with genotype, subgenotype and geographic region. We demonstrated heterogeneous prevalence of these mutations among HBV (sub)genotypes and locations, which may contribute to improvement of diagnostic procedures, immunization programs, therapeutic protocols, and disease prognosis. Since HBV (sub)genotypes and mutations are key determinants in the management and treatment of hepatitis B, reliable biomarkers and convenient methods need to be established to monitor viral factors and achieve a personalized treatment approach.

## Data Availability Statement

The original contributions presented in the study are included in the article/Supplementary Material, further inquiries can be directed to the corresponding author/s.

## Author Contributions

NA and NS: conceptualization. NA, ST, and NS: methodology, formal Analysis, and writing – review and editing. NA: writing – original draft preparation. All authors read and approved the final manuscript.

## Conflict of Interest

The authors declare that the research was conducted in the absence of any commercial or financial relationships that could be construed as a potential conflict of interest.

## Footnotes

1 https://unstats.un.org/unsd/methodology/m49/#geo-regions

## Supplementary Material

Supplementary Figure 1 Phylogenetic analysis of HBV complete genome sequences representative of genotypes A–J. Maximum likelihood phylogenetic tree of **(A)** 871 complete genome sequences of genotype A from HBVdb database plus 104 reference sequences; **(B)** 1755 complete genome sequences of genotype B from HBVdb database plus 104 reference sequences; **(C)** 2187 complete genome sequences of genotype C from HBVdb database plus 104 reference sequences; **(D)** 1059 complete genome sequences of genotype D from HBVdb database plus 104 reference sequences; **(E)** 249 complete genome sequences of genotype F from HBVdb database plus 104 reference sequences. (Sub)genotypes are indicated next to their respective clusters. Sequences whose subgenotype could not be determined were indicated as “not determined.” The numbers in branches indicate the statistical support (aLRT value). Only aLRT values ≥ 80 are shown.

Supplementary Table 1 HBV complete genome sequences dataset.

Supplementary Table 2 Clinically relevant HBV mutants by (sub)genotype and geographic region.
