# Supplementary figures and images for: Comprehensive Analysis of Clinically Significant Hepatitis B Virus Mutations in Relation to Genotype, Subgenotype and Geographic Region
Source: Front Microbiol. 2020 Dec 14;11:616023. doi: 10.3389/fmicb.2020.616023 (PMC7767914; doi:10.3389/fmicb.2020.616023)

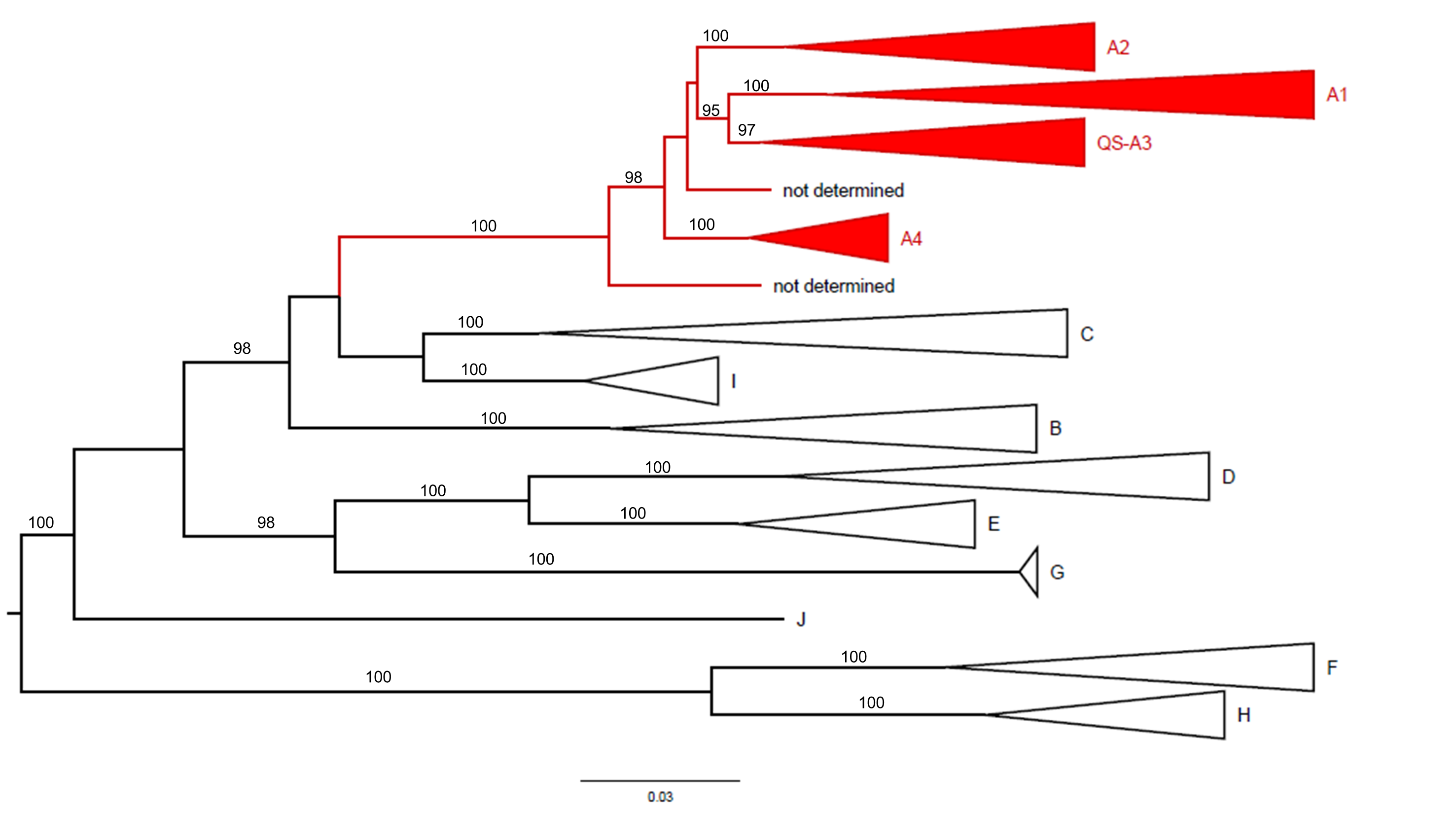

Supplement: Supplementary Figure 1 — Phylogenetic analysis of HBV complete genome sequences representative of genotypes A–J. Maximum likelihood phylogenetic tree of (A) 871 complete genome sequences of genotype A from HBVdb database plus 104 reference sequences; (B) 1755 complete genome sequences of genotype B from HBVdb database plus 104 reference sequences; (C) 2187 complete genome sequences of genotype C from HBVdb database plus 104 reference sequences; (D) 1059 complete genome sequences of genotype D from HBVdb database plus 104 reference sequences; (E) 249 complete genome sequences of genotype F from HBVdb database plus 104 reference sequences. (Sub)genotypes are indicated next to their respective clusters. Sequences whose subgenotype could not be determined were indicated as “not determined.” The numbers in branches indicate the statistical support (aLRT value). Only aLRT values ≥ 80 are shown. [file Image_1.TIF]

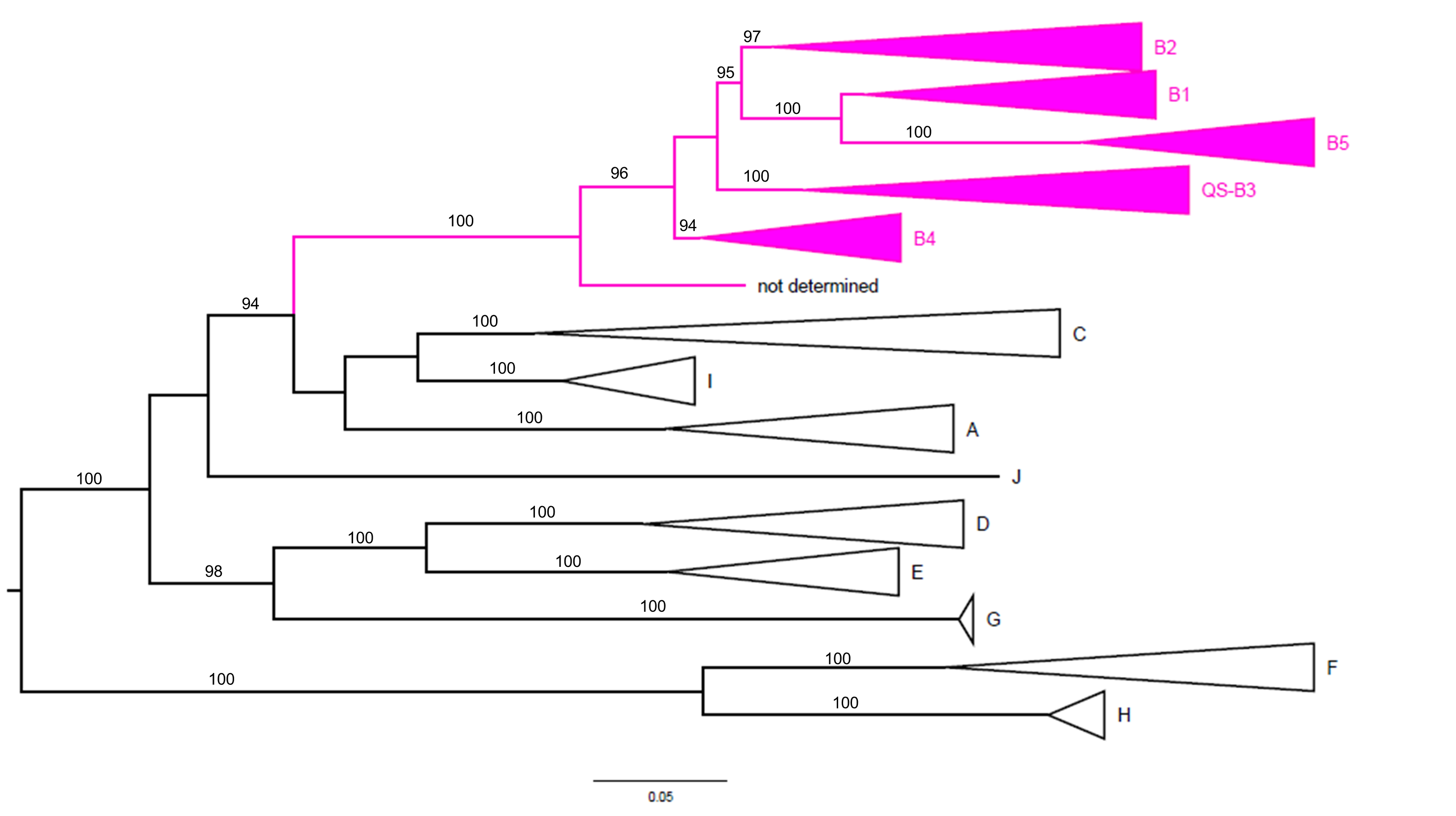

Supplement: Supplementary file 2 [file Image_2.TIF]

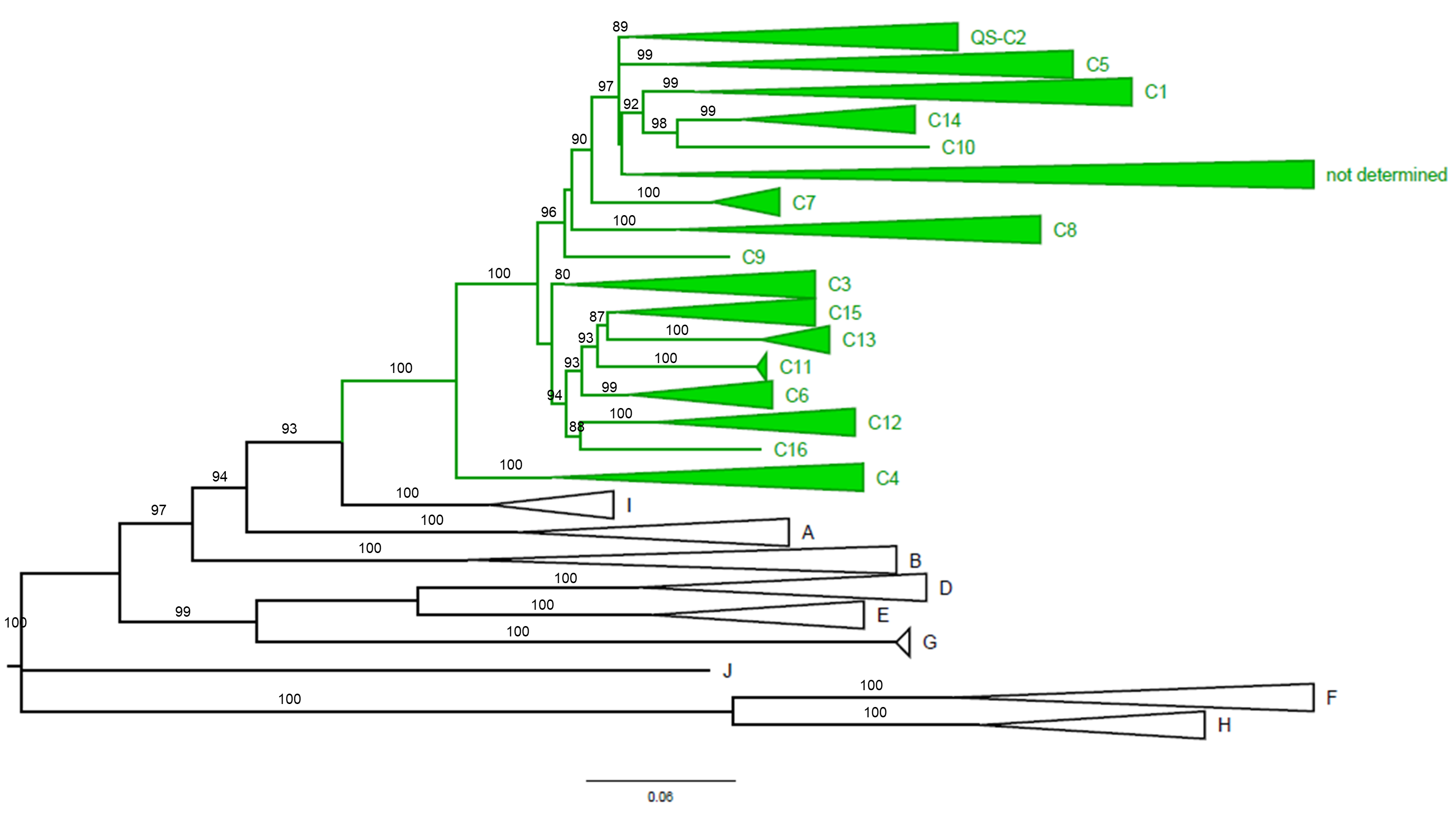

Supplement: Supplementary file 3 [file Image_3.TIF]

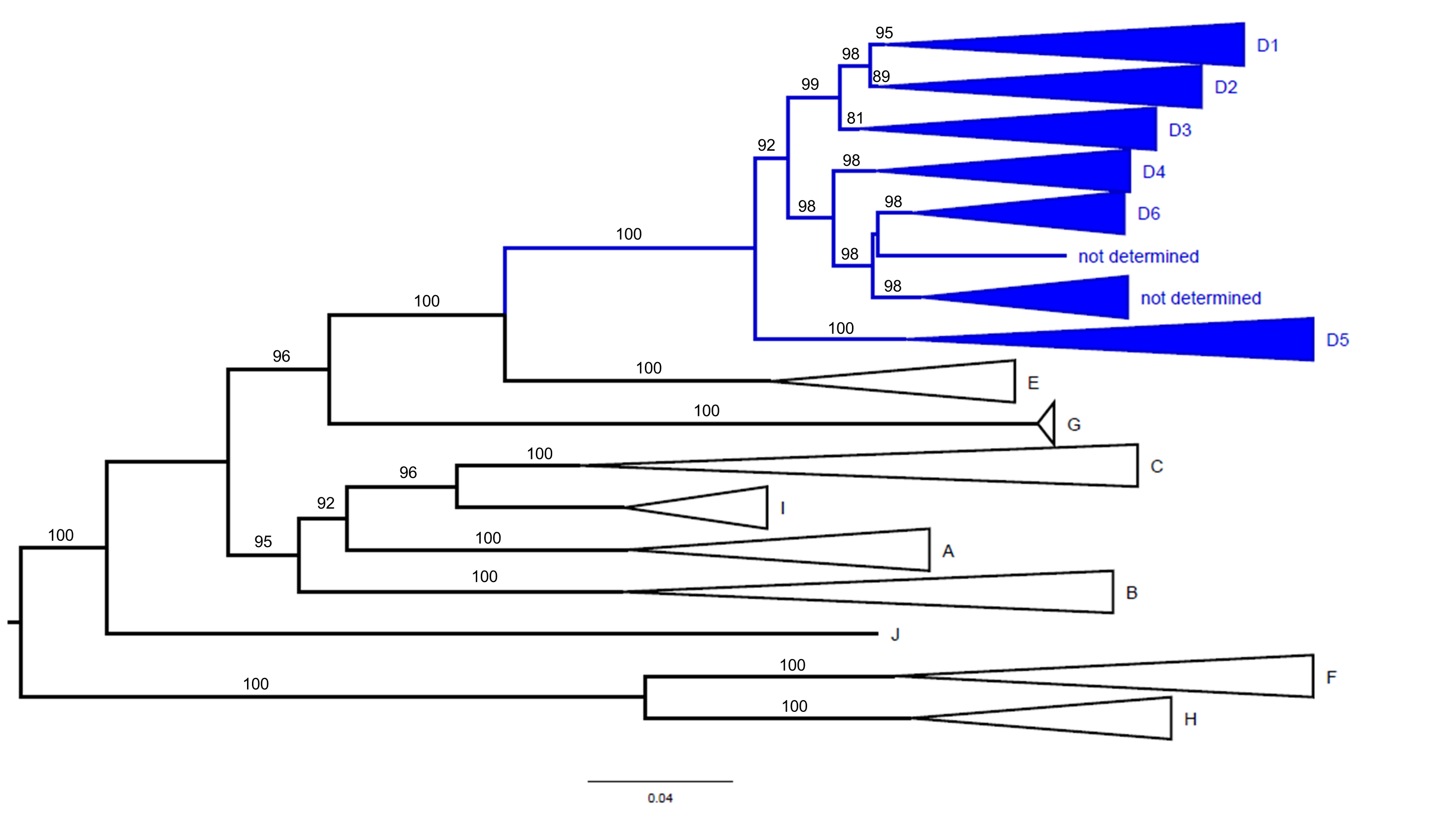

Supplement: Supplementary file 4 [file Image_4.TIF]

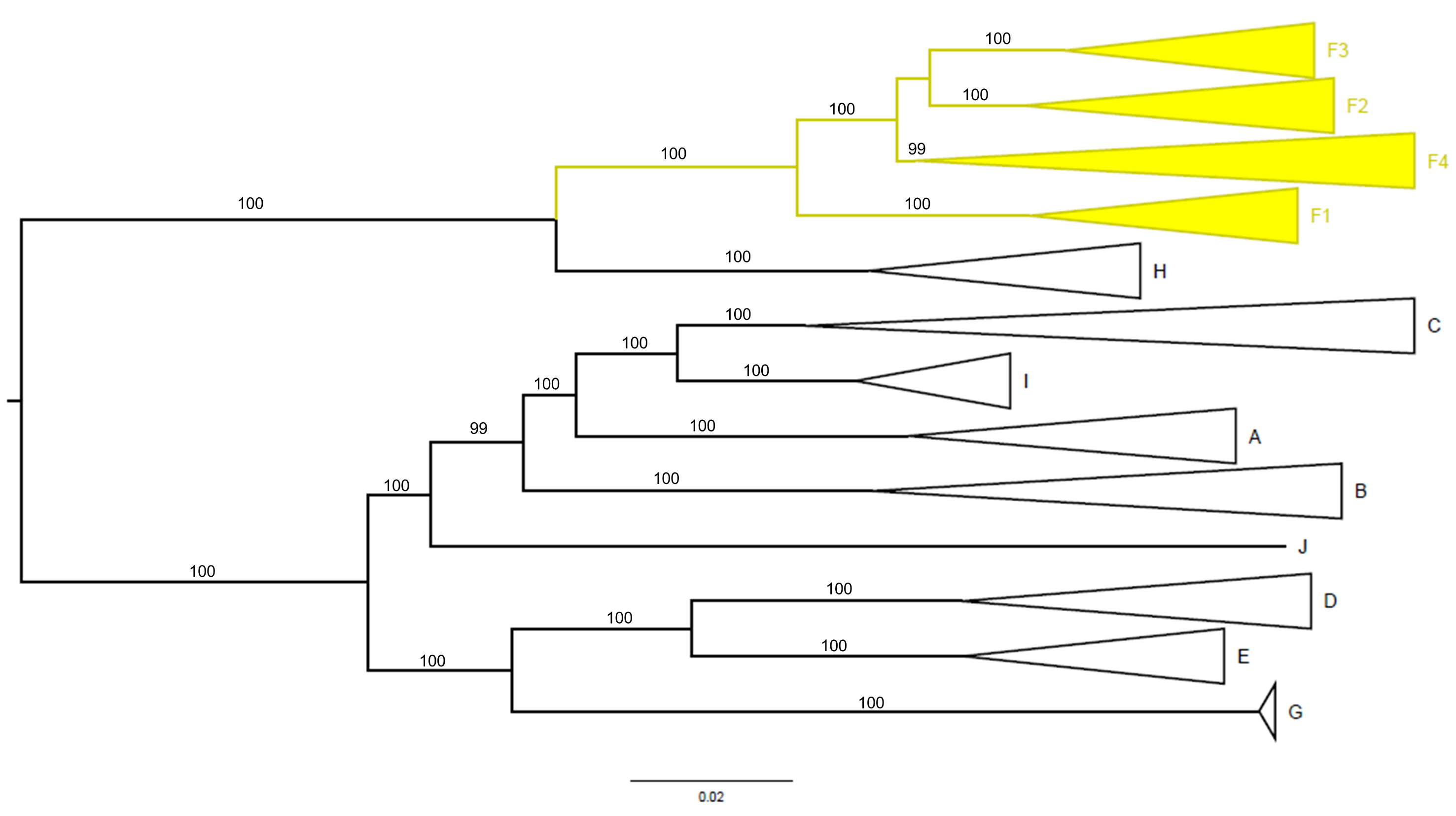

Supplement: Supplementary file 5 [file Image_5.TIF]
