# Supplementary material for: Comprehensive Analysis of Clinically Significant Hepatitis B Virus Mutations in Relation to Genotype, Subgenotype and Geographic Region
Source: Front Microbiol. 2020 Dec 14;11:616023. doi: 10.3389/fmicb.2020.616023 (PMC7767914; doi:10.3389/fmicb.2020.616023)
Supplement: Supplementary Table 2 — Clinically relevant HBV mutants by (sub)genotype and geographic region. [file Table_2.DOCX]

Table S2. Clinically relevant HBV mutants by (sub)genotype and geographic region

|  | Geographic Regions, n (%) | | | | | | | | | | | | | | | | | | | | |  |
| --- | --- | --- | --- | --- | --- | --- | --- | --- | --- | --- | --- | --- | --- | --- | --- | --- | --- | --- | --- | --- | --- | --- |
| Subgenotype  A1 | Australia and  New Zealand (n=0) | Caribbean  (n=28) | Central America (n=2) | Central Asia  (n=0) | Eastern Africa (n=60) | Eastern Asia  (n=8) | Eastern Europe  (n=0) | Melanesia  (n=0) | Middle Africa  (n=2) | Northern Africa (n=1) | North America (n=0) | Northern Europe (n=0) | Polynesia  (n=0) | South America (n=25) | South-eastern Asia (n=9) | Southern Africa (n=73) | Southern Asia (n=44) | Southern Europe (n=0) | Western Africa  (n=2) | Western Asia  (n=1) | Western Europe (n=4) | *P* |
| Escape mutants^a^ | NA | 0 | 0 | NA | 1 (1.7) | 0 | NA | NA | 0 | 0 | NA | NA | NA | 0 | 2 (22.2) | 4  (5.5) | 4  (9.1) | NA | 0 | 0 | 0 | 0.289 |
| HCC-associated mutants^b^ | NA | 9 (32.1) | 0 | NA | 15  (25) | 3 (37.5) | NA | NA | 1 (50) | 0 | NA | NA | NA | 6  (24) | 4 (44.4) | 16  (21.9) | 27 (61.4) | NA | 1  (50) | 0 | 1  (25) | 0.010 |
| LAM-resistant mutants^c^ | NA | 0 | 0 | NA | 1  (1.7) | 0 | NA | NA | 0 | 0 | NA | NA | NA | 0 | 0 | 0 | 0 | NA | 0 | 0 | 1  (25) | 0.001 |
|  | Geographic Regions, n (%) | | | | | | | | | | | | | | | | | | | | |  |
| Subgenotype  A2 | Australia and  New Zealand (n=0) | Caribbean  (n=3) | Central America (n=15) | Central Asia  (n=1) | Eastern Africa (n=0) | Eastern Asia (n=60) | Eastern Europe (n=43) | Melanesia  (n=0) | Middle Africa (n=0) | Northern Africa (n=0) | North America (n=248) | Northern Europe (n=8) | Polynesia  (n=0) | South America (n=22) | South-eastern Asia (n=0) | Southern Africa (n=5) | Southern Asia (n=0) | Southern Europe (n=6) | Western Africa (n=7) | Western Asia  (n=1) | Western Europe (n=107) | *P* |
| Escape mutants^a^ | NA | 0 | 0 | 0 | NA | 2  (3.3) | 1  (2.3) | NA | NA | NA | 41 (16.5) | 0 | NA | 1  (4.5) | NA | 0 | NA | 0 | 2  (28.6) | 0 | 4  (3.7) | 0.002 |
| HCC-associated mutants^b^ | NA | 0 | 0 | 0 | NA | 5  (8.3) | 18  (41.9) | NA | NA | NA | 100  (40.3) | 2  (25) | NA | 3  (13.6) | NA | 0 | NA | 4  (66.7) | 7  (100) | 0 | 10  (9.3) | < 0.001 |
| LAM-resistant mutants^c^ | NA | 0 | 1  (6.7) | 0 | NA | 1  (1.7) | 0 | NA | NA | NA | 103  (41.5) | 0 | NA | 0 | NA | 0 | NA | 0 | 0 | 0 | 12  (11.2) | < 0.001 |
|  | Geographic Regions, n (%) | | | | | | | | | | | | | | | | | | | | |  |
| Genotype  B | Australia and  New Zealand (n=0) | Caribbean  (n=0) | Central America (n=6) | Central Asia  (n=0) | Eastern Africa (n=0) | Eastern Asia (n=1220) | Eastern Europe (n=0) | Melanesia  (n=0) | Middle Africa (n=0) | Northern Africa (n=0) | North America (n=76) | Northern Europe (n=0) | Polynesia  (n=0) | South America (n=3) | South-eastern Asia (n=335) | Southern Africa (n=0) | Southern Asia (n=0) | Southern Europe (n=1) | Western Africa (n=0) | Western Asia  (n=0) | Western Europe (n=2) | *P* |
| Escape mutants^a^ | NA | NA | 1  (16.7) | NA | NA | 181  (14.8) | NA | NA | NA | NA | 3  (3.9) | NA | NA | 3  (100) | 47  (14) | NA | NA | 1  (100) | NA | NA | 0 | 0.031 |
| HCC-associated mutants^b^ | NA | NA | 1  (16.7) | NA | NA | 220  (18.0) | NA | NA | NA | NA | 16  (21.1) | NA | NA | 0 | 54  (16.1) | NA | NA | 0 | NA | NA | 1  (50) | 0.740 |
| LAM-resistant mutants^c^ | NA | NA | 0 | NA | NA | 32  (2.6) | NA | NA | NA | NA | 24  (31.6) | NA | NA | 0 | 2  (0.6) | NA | NA | 0 | NA | NA | 0 | < 0.001 |
|  | Geographic Regions, n (%) | | | | | | | | | | | | | | | | | | | | |  |
| Genotype  C | Australia and  New Zealand (n=15) | Caribbean  (n=0) | Central America (n=4) | Central Asia  (n=2) | Eastern Africa  (n=0) | Eastern Asia (n=1738) | Eastern Europe  (n=0) | Melanesia  (n=8) | Middle Africa  (n=0) | Northern Africa  (n=0) | North America (n=2) | Northern Europe (n=1) | Polynesia  (n=22) | South America  (n=3) | South-eastern Asia (n=267) | Southern Africa  (n=2) | Southern Asia  (n=42) | Southern Europe (n=0) | Western Africa  (n=0) | Western Asia  (n=0) | Western Europe  (n=2) | *P* |
| Escape mutants^a^ | 0 | NA | 0 | 0 | NA | 197  (11.3) | NA | 1  (12.5) | NA | NA | 0 | 0 | 1  (4.5) | 0 | 25  (9.4) | 0 | 6  (14.3) | NA | NA | NA | 0 | 0.905 |
| HCC-associated mutants^b^ | 3  (20) | NA | 2  (50) | 0 | NA | 877  (50.5) | NA | 2  (25) | NA | NA | 1  (50) | 0 | 6  (27.3) | 2  (66.7) | 116  (43.4) | 2  (100) | 24  (57.1) | NA | NA | NA | 0 | 0.019 |
| LAM-resistant mutants^c^ | 0 | NA | 0 | 0 | NA | 165  (9.5) | NA | 0 | NA | NA | 0 | 0 | 0 | 0 | 10  (3.7) | 0 | 2  (4.8) | NA | NA | NA | 0 | 0.001 |
|  | Geographic Regions, n (%) | | | | | | | | | | | | | | | | | | | | |  |
| Genotype  D | Australia and  New Zealand (n=36) | Caribbean  (n=10) | Central America (n=0) | Central Asia  (n=10) | Eastern Africa  (n=6) | Eastern Asia  (n=83) | Eastern Europe (n=96) | Melanesia  (n=5) | Middle Africa  (n=0) | Northern Africa (n=29) | North America (n=89) | Northern Europe (n=22) | Polynesia  (n=0) | South America (n=65) | South-eastern Asia (n=3) | Southern Africa (n=16) | Southern Asia (n=372) | Southern Europe (n=47) | Western Africa  (n=0) | Western Asia (n=122) | Western Europe (n=26) | *P* |
| Escape mutants^a^ | 4  (11.1) | 0 | NA | 0 | 0 | 5  (6.0) | 4  (4.2) | 0 | NA | 2  (6.9) | 26  (29.2) | 2  (9.1) | NA | 5  (7.7) | 0 | 0 | 33  (8.9) | 5  (10.6) | NA | 8  (6.6) | 2  (7.7) | < 0.001 |
| HCC-associated mutants^b^ | 7  (19.4) | 2  (20) | NA | 0 | 5  (83.3) | 31  (37.3) | 20  (20.8) | 1  (20) | NA | 6  (20.7) | 23  (25.8) | 3  (13.6) | NA | 18  (27.7) | 1  (33.3) | 4  (25) | 137  (36.8) | 14  (29.8) | NA | 42  (34.4) | 9  (34.6) | 0.005 |
| LAM-resistant mutants^c^ | 0 | 0 | NA | 0 | 0 | 1  (1.2) | 1  (1.0) | 0 | NA | 0 | 44  (49.4) | 0 | NA | 1  (1.5) | 0 | 0 | 13  (3.5) | 13  (27.7) | NA | 4  (3.3) | 1  (3.8) | < 0.001 |
|  | Geographic Regions, n (%) | | | | | | | | | | | | | | | | | | | | |  |
| Genotype  E | Australia and  New Zealand (n=0) | Caribbean  (n=0) | Central America (n=1) | Central Asia  (n=0) | Eastern Africa (n=3) | Eastern Asia  (n=0) | Eastern Europe (n=0) | Melanesia  (n=0) | Middle Africa (n=71) | Northern Africa (n=17) | North America (n=0) | Northern Europe (n=2) | Polynesia  (n=0) | South America (n=2) | South-eastern Asia (n=0) | Southern Africa (n=9) | Southern Asia (n=0) | Southern Europe (n=0) | Western Africa (n=160) | Western Asia  (n=2) | Western Europe (n=5) | *P* |
| Escape mutants^a^ | NA | NA | 0 | NA | 0 | NA | NA | NA | 0 | 0 | NA | 0 | NA | 0 | NA | 1  (11.1) | NA | NA | 11  (6.9) | 0 | 2  (40) | 0.022 |
| HCC-associated mutants^b^ | NA | NA | 1  (100) | NA | 0 | NA | NA | NA | 6  (8.5) | 4  (23.5) | NA | 1  (50) | NA | 0 | NA | 2  (22.2) | NA | NA | 53  (33.1) | 1  (50) | 1  (20) | 0.009 |
| LAM-resistant mutants^c^ | NA | NA | 0 | NA | 0 | NA | NA | NA | 1  (1.4) | 0 | NA | 0 | NA | 0 | NA | 0 | NA | NA | 0 | 0 | 0 | 0.970 |
|  | Geographic Regions, n (%) | | | | | | | | | | | | | | | | | | | | |  |
| Genotype  F | Australia and  New Zealand (n=0) | Caribbean  (n=0) | Central America (n=24) | Central Asia  (n=0) | Eastern Africa (n=0) | Eastern Asia  (n=2) | Eastern Europe (n=0) | Melanesia  (n=0) | Middle Africa  (n=0) | Northern Africa (n=0) | North America (n=8) | Northern Europe (n=1) | Polynesia  (n=0) | South America (n=213) | South-eastern Asia (n=0) | Southern Africa (n=0) | Southern Asia  (n=0) | Southern Europe (n=0) | Western Africa (n=0) | Western Asia  (n=0) | Western Europe (n=1) | *P* |
| Escape mutants^a^ | NA | NA | 1  (4.2) | NA | NA | 0 | NA | NA | NA | NA | 0 | 0 | NA | 4  (1.9) | NA | NA | NA | NA | NA | NA | 0 | 0.975 |
| HCC-associated mutants^b^ | NA | NA | 3  (12.5) | NA | NA | 2  (100) | NA | NA | NA | NA | 7  (87.5) | 0 | NA | 64  (30) | NA | NA | NA | NA | NA | NA | 0 | 0.001 |
| LAM-resistant mutants^c^ | NA | NA | 0 | NA | NA | 0 | NA | NA | NA | NA | 0 | 0 | NA | 8  (3.8) | NA | NA | NA | NA | NA | NA | 0 | 0.925 |
|  | Geographic Regions, n (%) | | | | | | | | | | | | | | | | | | | | |  |
| Genotype  G | Australia and  New Zealand (n=0) | Caribbean  (n=0) | Central America (n=7) | Central Asia  (n=0) | Eastern Africa (n=0) | Eastern Asia  (n=0) | Eastern Europe (n=0) | Melanesia  (n=0) | Middle Africa (n=0) | Northern Africa (n=0) | North America (n=16) | Northern Europe (n=1) | Polynesia  (n=0) | South America (n=7) | South-eastern Asia (n=1) | Southern Africa (n=1) | Southern Asia (n=0) | Southern Europe (n=0) | Western Africa (n=0) | Western Asia  (n=0) | Western Europe (n=4) | *P* |
| Escape mutants^a^ | NA | NA | 0 | NA | NA | NA | NA | NA | NA | NA | 2  (12.5) | 1  (100) | NA | 1  (14.3) | 0 | 0 | NA | NA | NA | NA | 0 | 0.126 |
| HCC-associated mutants^b^ | NA | NA | 7  (100) | NA | NA | NA | NA | NA | NA | NA | 16  (100) | 1  (100) | NA | 7  (100) | 0 | 1  (100) | NA | NA | NA | NA | 4  (100) | < 0.001 |
| LAM-resistant mutants^c^ | NA | NA | 0 | NA | NA | NA | NA | NA | NA | NA | 10  (62.5) | 0 | NA | 3  (42.9) | 0 | 0 | NA | NA | NA | NA | 0 | 0.043 |
|  | Geographic Regions, n (%) | | | | | | | | | | | | | | | | | | | | |  |
| Genotype  H | Australia and  New Zealand (n=0) | Caribbean  (n=0) | Central America (n=11) | Central Asia  (n=0) | Eastern Africa (n=0) | Eastern Asia  (n=7) | Eastern Europe (n=0) | Melanesia  (n=0) | Middle Africa (n=0) | Northern Africa (n=0) | North America (n=4) | Northern Europe (n=0) | Polynesia  (n=0) | South America (n=2) | South-eastern Asia (n=1) | Southern Africa (n=0) | Southern Asia (n=0) | Southern Europe (n=0) | Western Africa (n=0) | Western Asia  (n=0) | Western Europe (n=0) | *P* |
| Escape mutants^a^ | NA | NA | 0 | NA | NA | 0 | NA | NA | NA | NA | 0 | NA | NA | 0 | 0 | NA | NA | NA | NA | NA | NA | ̶ |
| HCC-associated mutants^b^ | NA | NA | 1  (9.1) | NA | NA | 1  (14.3) | NA | NA | NA | NA | 1  (25) | NA | NA | 1  (50) | 0 | NA | NA | NA | NA | NA | NA | 0.634 |
| LAM-resistant mutants^c^ | NA | NA | 1  (9.1) | NA | NA | 0 | NA | NA | NA | NA | 0 | NA | NA | 0 | 0 | NA | NA | NA | NA | NA | NA | 0.857 |

The *P*-value was listed as “ ̶ ” when the specific mutation was found in no genotype (statistical analysis not conducted). LAM, lamivudine; NA, Not available.

^a^Sequences with at least one immune escape mutation.

^b^Sequences with at least one HCC-associated mutation.

^c^Amino acid substitution profile for LAM resistance: rtM204V/I
